# Exdpf Is a Key Regulator of Exocrine Pancreas Development Controlled by Retinoic Acid and *ptf1a* in Zebrafish

**DOI:** 10.1371/journal.pbio.0060293

**Published:** 2008-11-25

**Authors:** Zhi Jiang, Jianbo Song, Fei Qi, An Xiao, Xizhou An, Ning-ai Liu, Zuoyang Zhu, Bo Zhang, Shuo Lin

**Affiliations:** 1 Key Laboratory of Cell Proliferation and Differentiation, Center of Developmental Biology and Genetics, College of Life Sciences, Peking University, Ministry of Education, Beijing, People's Republic of China; 2 Department of Molecular, Cell, and Developmental Biology, University of California, Los Angeles, Los Angeles, California, United States of America; 3 Department of Medicine, Cedars-Sinai Research Institute, University of California, Los Angeles, Los Angeles, California, United States of America; Wellcome Trust Sanger Institute, United Kingdom

## Abstract

Both endocrine and exocrine pancreatic cells arise from *pancreatic-duodenal homeobox 1* (*pdx1*)-positive progenitors. The molecular mechanisms controlling cell fate determination and subsequent proliferation, however, are poorly understood. Unlike endocrine cells, less is known about exocrine cell specification. We report here the identification and characterization of a novel exocrine cell determinant gene, *exocrine differentiation and proliferation factor* (*exdpf*), which is highly expressed in the exocrine cell progenitors and differentiated cells of the developing pancreas in zebrafish. Knockdown of *exdpf* by antisense morpholino caused loss or significant reduction of exocrine cells due to lineage-specific cell cycle arrest but not apoptosis, whereas the endocrine cell mass appeared normal. Real-time PCR results demonstrated that the cell cycle arrest is mediated by up-regulation of cell cycle inhibitor genes *p21^Cip^*, *p27^Kip^*, and *cyclin G1* in the *exdpf* morphants. Conversely, overexpression of *exdpf* resulted in an overgrowth of the exocrine pancreas and a severe reduction of the endocrine cell mass, suggesting an inhibitory role for *exdpf* in endocrine cell progenitors. We show that *exdpf* is a direct target gene of pancreas-specific transcription factor 1a (Ptf1a), a transcription factor critical for exocrine formation. Three consensus Ptf1a binding sites have been identified in the *exdpf* promoter region. Luciferase assay demonstrated that Ptf1a promotes transcription of the *exdpf* promoter. Furthermore, *exdpf* expression in the exocrine pancreas was lost in *ptf1a* morphants, and overexpression of *exdpf* successfully rescued exocrine formation in *ptf1a*-deficient embryos. Genetic evidence places *expdf* downstream of retinoic acid (RA), an instructive signal for pancreas development. Knocking down *exdpf* by morpholino abolished ectopic *carboxypeptidase A* (*cpa*) expression induced by RA. On the other hand, *exdpf* mRNA injection rescued endogenous *cpa* expression in embryos treated with diethylaminobenzaldehyde, an inhibitor of RA signaling. Moreover, exogenous RA treatment induced anterior ectopic expression of *exdpf* and trypsin in a similar pattern. Our study provides a new understanding of the molecular mechanisms controlling exocrine cell specification and proliferation by a novel gene, *exdpf*. Highly conserved in mammals, the expression level of *exdpf* appears elevated in several human tumors, suggesting a possible role in tumor pathogenesis.

## Author Summary

The pancreas is a vital organ comprising endocrine and exocrine components. Both endocrine and exocrine cells derive from a common pool of progenitors present in the gut endoderm during embryogenesis. The molecular mechanisms regulating cell fate decisions and lineage-specific proliferation are not fully understood. In this work, we report the characterization of a novel gene, *exocrine differentiation and proliferation factor* (*exdpf*), as a regulator for exocrine cell fate and differentiation/proliferation. We show that it is a direct target of the transcription factor pancreas-specific transcription factor 1a (Ptf1a), which is expressed in progenitors that give rise to all pancreatic cell types. We find that a deficiency of *exdpf* results in a severe reduction of exocrine size due to defects in cell proliferation. Consistent with this finding, overexpression of *exdpf* leads to an increase of exocrine size and a decrease of endocrine size, suggesting a possible change in fate of the endocrine progenitors. The human ortholog of *exdpf* is highly conserved and its expression level appears elevated in several cancers, including hepatic and pancreatic cancers, implying a possible role in pathogenesis of these malignancies.

## Abstract 3

The zebrafish*exdpf*, a novel regulator of pancreatic exocrine cell fate, is essential for exocrine cell differentiation and proliferation.

## Introduction

The pancreas is a mixed organ with endocrine and exocrine compartments. The endocrine portion contains four distinct hormone-producing cell types organized into islets of Langerhans. Autoimmune-mediated destruction of endocrine β cells causes type 1 diabetes [1,2]. β cell number also gradually declines in type 2 diabetes [2]. The exocrine portion includes acinar cells, which produce digestive enzymes, and duct cells, which form an elaborate duct system that transports these enzymes into the gut. The majority of malignant pancreatic cancers derive from the exocrine portion [3]. Development of all major pancreatic cell types, including endocrine, exocrine, and duct cells, requires the function of the *pancreatic-duodenal homeobox 1* (*Pdx1,* also known as *Ipf-1*) gene [4,5]. The molecular mechanisms determining early cell fate and the subsequent proliferation of endocrine and exocrine cells are not fully understood. Identification and characterization of novel lineage-specific regulators of exocrine pancreas cell proliferation could shed light on the pathogenesis of pancreatic cancers.

Morphogenesis of the pancreas in zebrafish (Danio rerio) shares some similarities to that in the mouse. In mice, the pancreas develops from one dorsal and one ventral bud that arise from the posterior foregut [6–8] sequentially. The recognizable dorsal pancreatic bud forms from the prepatterned endoderm at around 22–25 somites (embryonic day 9.5 [E9.5]) and the ventral bud arises slightly later at around 30 somites (E10.25 to E10.5). Then the dorsal and ventral buds fuse as a result of gut rotation at E12.5 [9]. Different endocrine cell types are specified at different stages. The α and β cells mature at E9.5 since *glucagon* and *preproinsulin* can be detected by immunohistochemistry [10], whereas somatostatin can be detected only at E13.5 [11,12]. Initially, it had been thought that the zebrafish pancreas develops from a single pancreatic anlage that appears at around 15 h postfertilization (hpf) [13–15]. This posterodorsal pancreatic anlage gives rise only to endocrine cells. Using a gut:GFP transgenic line, however, Field et al. observed a second anlage (ventral anlagen) that arose from the foregut at 34 hpf [16] when exocrine cells begin differentiation. In addition to exocrine cells, this anteroventral anlage also contributes to endocrine cells that are scattered outside of the main islet [16].

The dynamic process of pancreatic development is controlled by extrinsic signals from the adjacent tissues and intrinsic transcription factors. Multiple signals including fibroblast growth factor [17,18], bone morphogenetic protein [19], Notch [17,20–22], and sonic hedgehog [23] play critical roles for proper pancreas formation. A conserved role of retinoic acid (RA) has been reported in many organisms, including zebrafish [24,25], *Xenopus* [26], and mouse [27,28]. There are conflicting data, however, on the relative effects of RA on endocrine and exocrine pancreas differentiation. In *Xenopus*, RA treatment promotes endocrine at the expense of exocrine differentiation in the dorsal bud by inhibiting Notch signaling activity [26]. In zebrafish, RA treatment results in anterior expansion of endocrine and exocrine cells [25]. It appears that RA acts directly in the endoderm to induce endocrine pancreatic precursors [29]. In mouse embryonic pancreas cultures, *all-trans* retinoic acid (*at*RA) inhibits branching morphogenesis and exocrine cell differentiation but accelerates endocrine differentiation, possibly due to increased level of *Pdx1* in the endocrine clusters [30]. The differential effects may be explained by the distribution of the RAR and RXR receptors in the developing mouse pancreas [31].

A network of intrinsic transcription factors that act in a cascade fashion to initiate and maintain cell-specific gene expression patterns determines the ultimate lineage-specific cell fate. One of the earliest transcription factors functioning in the developing pancreatic epithelium is PDX1, which plays an essential role during the early phase of pancreas development. Mice with a targeted mutation in the *Pdx1* gene exhibited no development of pancreatic tissue [4]. The agenesis of the pancreas is caused by an early arrest right after initial bud formation [4,5]. Furthermore, multiple roles of *Pdx1* in cell lineage determination during pancreas formation has been revealed by lineage tracing using a modified version of Cre/lox technology [32]. Cells labeled between E9.5 and E11.5 give rise to all three pancreatic cell lineages including islet, exocrine acini, and ducts. Conversely, cells labeled at E8.5 and E.12.5 or thereafter give rise only to endocrine and acinar cells [32]. These results suggest that temporal regulation of *Pdx1* expression is critical for cell fate determination. In addition, several transcription factors have been identified as endocrine specific determinants. Neurogenin 3 (Ngn3) is one of the most important endocrine specific transcription factors [20,33]. In contrast to the endocrine lineage, little is known about the mechanisms that control the differentiation of exocrine and ductal lineages. Initially, pancreatic transcription factor 1, alpha subunit (Ptf1a) had been considered an exocrine specific transcription factor since its expression becomes restricted to exocrine cells by E13.5 in mice [34]. However, cell lineage tracing experiments revealed that *ptf1a*-expressing cells give rise to all pancreatic cell types [35].

Here, we provide several lines of evidence indicating that *exocrine differentiation and proliferation factor* (*exdpf*), a novel gene identified from zebrafish but highly conserved in mouse and human, is an exocrine cell determinant and required regulator of cell proliferation. The protein encoded by *exdpf* is a putative signaling molecule and is expressed highly in the exocrine cells during pancreas formation in zebrafish. Knocking down *exdpf* by antisense morpholino caused significant reduction or loss of expression of exocrine markers. In contrast, overexpression of *exdpf* resulted in the overgrowth in size of the exocrine pancreas and remarkable decrease of endocrine cells. This result suggests that misexpression of *exdpf* in the endocrine precursors is able to transform their fate. We further show that the reduction of exocrine cells in *exdpf* morphants is due to lineage-specific cell cycle arrest. Real-time PCR revealed that the expressions of cell cycle inhibitor genes *p21^Cip^* and *p27^Kip^* are dramatically increased in the *exdpf* morphants. To test the effect of RA on exocrine cell differentiation, we performed an epistatic study of *exdpf* and the RA pathway. The results show that *exdpf* acts genetically downstream of RA. Exogenous RA treatment induced anterior ectopic exocrine cells via *exdpf* induction. Moreover, injection of *exdpf* mRNA partially restored exocrine cell differentiation at the endogenous area in embryos treated with exogenous RA, whereas the expansion of endocrine cells were reduced. Our data establish a critical role of *exdpf* in exocrine cell fate determination and proliferation. Being a vertebrate-specific gene, the *exdpf* orthologs are highly conserved from fish to human. A search of National Center for Biotechnology Information (NCBI) database revealed that the human *exdpf* ortholog expression is up-regulated in several human cancers including hepatic, pancreatic, and renal cancers, suggesting that overexpression or mutation in the *exdpf* gene might be involved in the pathogenesis of cancers.

## Results

### Vertebrate Orthologs of the *exdpf* Gene Are Highly Conserved

In our effort to identify pancreas specific genes, we isolated the *exdpf* gene (GeneID: 338304 [http://www.ncbi.nlm.nih.gov/sites/entrez]) from a RNA whole-mount in situ hybridization screen in zebrafish (our unpublished data). A BLAST search of the zebrafish genome revealed a homolog of *exdpf* named *endocrine differentiation and proliferation factor* (*endpf,* not described here). The deduced peptide encoded by the *exdpf* gene contains 117 amino acids. Multiple sequence alignments using ClustalW showed that the Exdpf protein is specific to vertebrates and highly conserved across the vertebrates including zebrafish, mouse, and human (Figure S1A). The human and mouse orthologs are known as uncharacterized novel open reading frames (*c20orf149,* Gene ID: 79144 and AK154758*)*. Overall, the deduced protein is about 42% identical across different species. The N terminus is highly conserved whereas the C terminus is more diversified, which suggests that subtle functional differences might lie in the C terminus. In addition, synteny analysis showed that a cluster of homologous genes is also conserved between zebrafish and human at the *exdpf* loci (Figure S1B).

### The *exdpf* Gene Is Highly Expressed in the Exocrine Cells during Pancreas Formation in Zebrafish

We studied the temporal and spatial expression of *exdpf* by reverse transcriptase PCR (RT-PCR) and whole-mount in situ hybridization. RT-PCR results (Figure S2) indicated that the *exdpf* transcript is maternally deposited since it was detected at the one-cell stage. The amount of *exdpf* transcript reduced following the one-cell stage, and the lowest level was detected at the shield stage. Then *exdpf* expression gradually increased from shield stage and a strong level was detected between 1 d postfertilization (dpf) and 2 dpf when the exocrine pancreas starts to develop; the highest level of expression was detected between 2 dpf and 5 dpf, the longest time point of this study. This result suggests that zygotic expression of the *exdpf* gene starts at around the shield stage.

**Figure 1:**
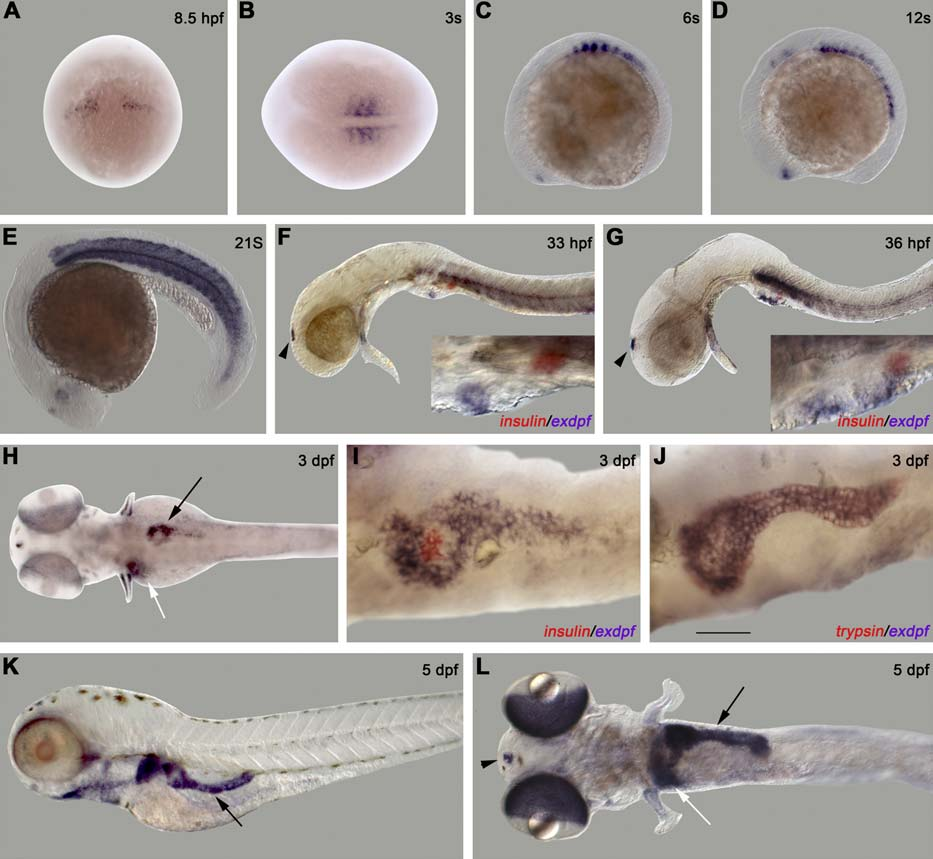
Spatiotemporal Expression of *exdpf* during Zebrafish Embryogenesis (A–L) In situ hybridization using an *exdpf* probe or an *exdpf* probe in combination with an *preproinsulin* probe (F, G and I) or a *trypsin* probe (J). (A) 8.5 hpf, dorsal view with anterior on the top. (B) 3-somite, dorsal view with anterior to the left. (C-G) Lateral view with anterior to the left. (C) 6-somite. (D) 12-somite. (E) 21-somite. Note strong expression in muscle area. (F) 33 hpf. (G) 36 hpf. In (F and G): Black arrowhead: epiphysis. Blue: *exdpf* probe; Red: *preproinsulin* probe. Inset: enlargement of pancreatic area. (H-J) Dorsal view with anterior to the left. (H) 3 dpf. Black arrow: pancreas. White arrow: liver. (I) 3 dpf. Double in situ hybridization using an *exdpf* probe (purple) and an *preproinsulin* probe (red). Note *exdpf* positive cells encircle *preproinsulin*-expressing cells; but *exdpf* is not expressed in those cells. (J) 3 dpf. Double in situ hybridization using an *exdpf* probe (blue) and a *trypsin* probe (red). Note that *exdpf* and *trypsin* perfectly overlap. (K, L) 5 dpf. (K) Lateral view with anterior to the left. Black arrow: pancreas. (L) Dorsal view with anterior to the left. Black arrow: pancreas. White arrow: liver. Black arrowhead: epiphysis. Scale bar for (I, J): 50 μm.

We then performed RNA whole-mount in situ hybridization using an *exdpf* probe to obtain detailed expression analysis of *exdpf* in the developing pancreas. Double in situ hybridization was performed using either a *preproinsulin* probe to locate the endocrine β cells or a *trypsin* probe to mark the exocrine cells in combination with the *exdpf* probe. *Exdpf* transcripts were first detected in the developing somites at 8.5 hpf (Figure 1A). From the three-somite to 21-somite stage, *exdpf* was expressed in somites, adaxial cell, slow muscle fiber, and epiphysis (Figure 1B–1E). Interestingly, *exdpf* started to express in the pancreatic area at 33 hpf (Figure 1F), just before exocrine specification begins. Later in development, the strongest domain of *exdpf* expression appeared in the pancreas. Cells expressing *exdpf* (blue staining in Figure 1F) were located about one somite anterior to the cluster of *preproinsulin*-positive cells (red staining in Figure 1F). By 36 hpf, *exdpf*-expressing cells started to contact the *preproinsulin*-positive cells (Figure 1G) as a result of gut rotation. By 2 dpf, *exdpf*-expressing cells embraced the cluster of *preproinsulin*-positive cells (unpublished data). These *exdpf*-expressing cells continue to grow posteriorly to form a typical pancreas-like shape at 3 dpf (Figure 1H–1J). From 33 hpf to 3 dpf, there was no overlap between *exdpf*-expressing cells and *preproinsulin*-positive cells, indicating that *exdpf* expression is excluded from endocrine cells. Conversely, *exdpf* transcripts completely overlap with *trypsin* expression at 3 dpf (Figure 1J). To confirm the exocrine-specific expression of *exdpf* at 4 dpf, we performed a double in situ hybridization using an *exdpf* probe and a probe against *carboxypeptidase A* (*cpa*), another exocrine marker. As expected, *exdpf* expression completely overlaps with *cpa* expression at 4 dpf (unpublished data). Together, these data suggest that *exdpf* is expressed exclusively in the exocrine cells during pancreas development.

### Reduced *exdpf* Impairs Exocrine Cell Differentiation and Growth

**Figure 2:**
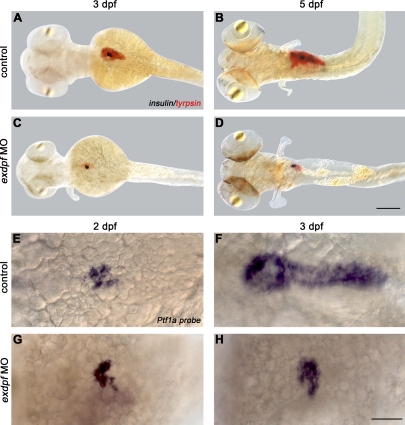
*Exdpf* Is Required for the Exocrine Pancreas Differentiation and Expansion (A–D) Double in situ hybridization using a *trypsin* probe (red) and a *preproinsulin* probe (blue). (A and B) Wild type (WT) embryos. (C and D) Embryos injected with *exdpf* morpholino. (A) A 3 dpf embryo. Note exocrine pancreas already expanded. (B) A 5 dpf embryo. Note both endocrine and exocrine mass increased. (C) An example of *exdpf* morphants, 3 dpf. Note exocrine mass is restricted to the anterior area, whereas endocrine portion appears normal. (D) An example of *exdpf* morphant at 5 dpf. Note exocrine pancreas is still restricted to the anterior area with limited increase in mass. All embryos are mounted in dorsal view, anterior to the left. Scale bar: 100 μm. (E–H) In situ hybridization using a *Ptf1a* probe. Dorsal view, anterior to the left. (E and F) Wild type embryos at 2 dpf and 3 dpf, respectively. (G and H) Examples of *exdpf* morphants at 2 dpf and 3 dpf, respectively. Note *Ptf1a* expression is restricted to the anterior area at 2 dpf (G); the expression is still limited in the anterior area and the mass does not increase at 3 dpf (H). Scale bar: 50 μm.

Based on the expression pattern of *exdpf*, we postulated that it is required for exocrine pancreas development. To test this hypothesis, we knocked down *exdpf* by injection of antisense morpholino oligonucleotides (MO1*^exdpf^* and MO2*^exdpf^*) designed to interfere with its translation. We tested both morpholinos to assure that the phenotypes observed are due to the specific knockdown of *exdpf*. A morpholino standard control oligonucleotide from Gene Tools was used to inject the control embryos. No specific phenotypes were observed in these control embryos. Double in situ hybridization using a *trypsin* probe and a *preproinsulin* probe was performed to assess the effect on exocrine and endocrine development simultaneously. In the control embryos, β cells formed a cluster (islet) that was surrounded by exocrine cells at the anterior area of the pancreas (head) at 3 dpf (Figure 2A). Both the β cell mass and exocrine mass increased at 5 dpf in the control embryos (Figure 2B). In addition, exocrine cells expanded posteriorly to form a typical pancreas like shape (Figure 2A and 2B). The majority of embryos injected with 2 ng of *exdpf* morpholino (MO1*^exdpf^*) exhibited no *trypsin* expression at 3 dpf (86%, *n* = 50) or 5 dpf. However, there were a few embryos (14%, *n* = 50) with reduced *exdpf* function that showed severe reduction of *trypsin* expression at 3 dpf and the remaining exocrine cells were restricted to the anterior pancreatic area engulfing the endocrine cells (Figure 2C). Furthermore, the exocrine cells failed to grow and expand posteriorly by 5 dpf (Figure 2D). In contrast, the endocrine cells looked largely normal in *exdpf* morphants (Figure 2C and 2D). Only a small fraction of *exdpf* morphants (10%, *n* = 50) showed scattered *preproinsulin* cells at 5 dpf (unpublished data). These results indicate that *exdpf* is required specifically for exocrine cell differentiation and growth. Over 90% of MO2*^exdpf^* morphants exhibited similar phenotypes (Figure S3). To investigate the function of *exdpf* in the differentiated exocrine cells, we used MO1*^exdpf^* for the rest of this study because it gave milder phenotypes.

We then studied early exocrine differentiation using a *ptf1a* probe. The protein encoded by *ptf1a* is a basic helix-loop-helix (bHLH) transcription factor that plays a critical role in exocrine pancreas development. A null mutation of *Ptf1a* in mouse leads to complete agenesis of exocrine pancreas and spatially disorganized endocrine pancreas [34,35]. In zebrafish, *ptf1a* loss of function by antisense morpholino injection suppresses exocrine markers without affecting endocrine markers and the organization of the main islet [36,37]. Since *ptf1a* can serve as an early marker of exocrine development [36], we analyzed *ptf1a* expression in *exdpf* morphants at 2 dpf and 3 dpf by whole-mount in situ hybridization. In the control embryos, *ptf1a*-expressing cells formed a loose cluster at 2 dpf (Figure 2E) and the expression expanded toward the posterior with exclusion from the endocrine cells at 3 dpf (Figure 2F). Expression of *ptf1a* was missing in the vast majority of *exdpf* morphant embryos (85%, *n* = 50). In a small fraction of *exdpf* morphants (15%, *n* = 50), initial expression of *ptf1a* appeared normal at 2 dpf (Figure 2G). But the expression of *ptf1a* failed to expand towards the posterior by 3 dpf (Figure 2H) and remained in the same area as in 2 dpf. This result confirms that *exdpf* is critical for exocrine cell specification and expansion.

The *exdpf* gene is expressed in the developing somites as well as the exocrine pancreas (Figure 1). To clarify whether the exocrine pancreas defect is due to pleiotropic abnormalities, we used a recently obtained transgenic fish MP760GFP (our unpublished data) that expresses green fluorescent protein (GFP) in the developing liver, gut, and pancreas. In this transgenic line, pancreatic expression of GFP is restricted in the exocrine portion. A minimal amount of *exdpf* morpholino was used to achieve the least amount of defects in overall body morphology. At 24 hpf, strong expression of GFP was observed in the presumed pancreatic area in both control embryos and *exdpf* mRNA injected embryos (Figure S5A and S5B, arrows). By 2 dpf, pancreatic GFP expression became more obvious in the control and *exdpf* mRNA-injected embryos (Figure S5A and S5B, arrowheads). In contrast, only residual or no pancreatic GFP expression was observed in *exdpf* morphants (Figure S5C). However, gut GFP expression in *exdpf* morphants (Figure S5C) remained comparable to that in the control embryos (Figure S5A).

*Exdpf* is also expressed in the developing liver (Figure 1) during embryogenesis. To assess whether it is required for liver development, in situ hybridization was performed using a *ceruloplasmin* (*cp*) probe. At 3 dpf, clear expression of *cp* was detected in the livers of control embryos injected with standard morpholino control (Figure S5D). No detectable change of *cp* expression was observed in *exdpf* morphants (Figure S5E–S5G). This might be due to the redundant function of *exdpf* homolog *endpf* since it is also expressed in the developing liver.

### The *exdpf* Gene Is a Direct Target of *ptf1a*

**Figure 3:**
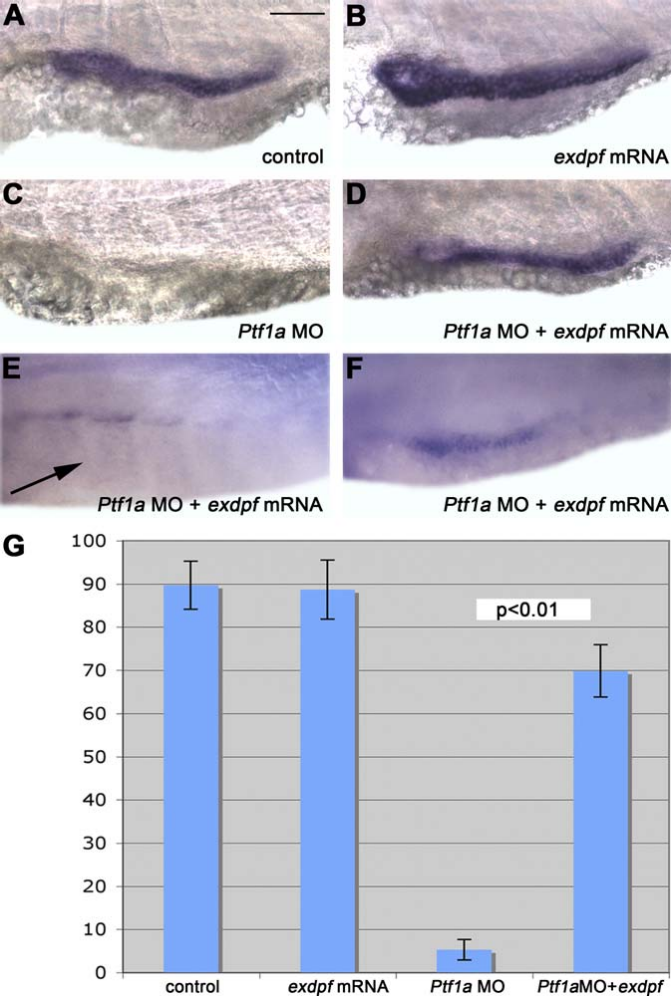
*Exdpf* Restores Expression of the Exocrine Cell Marker in *Ptf1a* Morphants (A–C) In situ hybridization using a *carboxypeptidase A* (*cpa*) probe in 3 dpf embryos. (A) A control embryo injected with water. (B) An embryo injected with 100 pg of *exdpf* mRNA. Note enhanced expression of *cpa*. (C) An embryo injected with 2 ng of *Ptf1a* morpholino. No expression of *cpa* was detected. (D–F) Embryos injected with 100 pg of *exdpf* mRNA followed by 2 ng of *Ptf1a* morpholino. (D) Almost full restoration of *cpa* expression. (E) No detectable *cpa* expression in the presumed exocrine area (arrow). (F) Partial restoration of *cpa* expression. All embryos are shown in lateral view, anterior to the left. Scale bar: 50 μm. (G) Percentage of embryos with *cpa* expression at 3 dpf. Y-axis represents mean ± SD.

We further studied the genetic interaction between *ptf1a* and *exdpf*. In mild *exdpf* morphants (injected with 2 ng of MO1*^exdpf^*), *ptf1a* expression was initiated but restricted to the anterior area in 72% of embryos (Figure 2G, *n* = 90), indicating that exocrine cell differentiation can start but expansion fails. However, only 15% of embryos (*n* = 50) still exhibited *ptf1a* expression when injected with 4 ng of MO1*^exdpf^*, suggesting a role for *exdpf* in exocrine cell differentiation. Knocking down *ptf1a* by morpholino injection (Figure 3) resulted in agenesis of the exocrine pancreas (Figure 3C); only about 5% of *ptf1a* morphants exhibited weak *cpa* expression (*n* = 189, Table 1). In the control embryos, injection of *exdpf* mRNA led to a great expansion of the exocrine pancreas (Figure 3B). Interestingly, injection of *exdpf* mRNA into *ptf1a* morphants successfully restored expression of exocrine marker *cpa* to about 70% of embryos (Figure 3D–3F, *n* = 168). About 35% of embryos exhibited nearly full restoration of *cpa* expression in the *ptf1a* morphants when *exdpf* mRNA was injected, whereas another 35% exhibited partial restoration (Table 1). In a reciprocal experiment, injection of *ptf1a* mRNA into *exdpf* morphants failed to rescue expression of exocrine markers (unpublished data). Together, these results place *exdpf* genetically downstream of *ptf1a* in exocrine development.

**Table 1:**
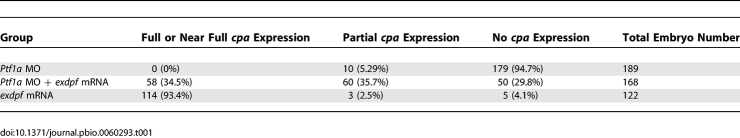
Quantitative Data of *cpa* Expression in *Ptf1a* Morphants

**Figure 4:**
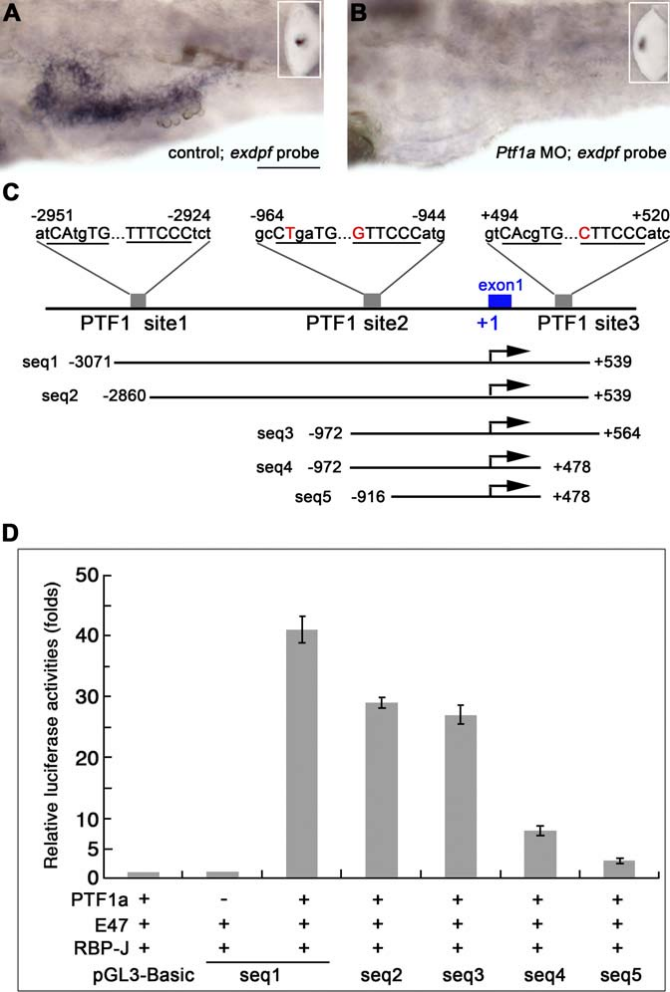
*Exdpf* Expression Is Regulated by Ptf1a (A and B) In situ hybridization using an *exdpf* probe in 3 dpf embryos. (A) An embryo injected with standard morpholino control. Inset: epiphysis expression. (B) An embryo inject with *Ptf1a* morpholino. Inset: epiphysis expression. Note that no exocrine expression of *exdpf* was detected. All embryos are shown in lateral view, anterior to the left. Scale bar: 50 μm. (C) Locations and sequences of the three potential PTF1 binding sites in zebrafish *exdpf* gene and different *exdpf* promoter segments used in luciferase assay. The E-box and TC-box are underlined and capital letters indicate conserved nucleotides. The nucleotides that are not confirmed to the conserved nucleotides are in red. Dots indicate the spaces between the E-box and TC-box. (D) Comparison of the activities of the segments containing different Ptf1 binding sites when transfected into 293 cells. These segments were inserted just before the ORF of luciferase gene. Luciferase reporter activity was adjusted for transfection efficiency and expressed relative to the promoterless pGL3-Basic vector. Error bars represent standard deviations.

To test whether *ptf1a* functions through *exdpf* in exocrine specification, we performed in situ hybridization analysis of *exdpf* in *ptf1a* morphants (Figure 4). Indeed, *exdpf* expression in the exocrine pancreas was abolished in such embryos, as expected (Figure 4B). However, the epiphysis expression of *exdpf* remained unchanged in the *ptf1a* morphants (Figure 4B, inset), indicating that *ptf1a* specifically controls pancreatic expression of *exdpf*. Ptf1 is an unusual heterotrimeric bHLH transcription factor composed of Ptf1a/P48, a common class A bHLH protein (such as HEB, E2–2, E12, or E47, also called E-proteins), and a third protein that can be either the mammalian Suppressor of Hairless RBP-J or its paralog, RBP-L [38,39]. Ptf1 binding sites are bipartite with an E-box (CANNTG) and a TC-box (TTTCCC) spaced one or two helical turns apart, center to center [39–41]. The heterodimeric subcomplex of Ptf1a and the E-protein binds to the E-box and RBP-J or RBP-L binds to the TC-box [39,41]. Binding of the Ptf1 complex to DNA requires both boxes, and the spacing between these elements is critical for Ptf1 binding [42]. Functional binding sites for the Ptf1 complex are present in the 5′ promoter regions of all of the acinar digestive enzyme genes examined [40,41]. To determine whether Ptf1a might indeed control the transcription of *exdpf*, we searched the 5-kb 5′ flanking region and intronic sequences of this gene for potential PTF1-binding sites comprising an E-box and a TC-box spaced one or two helical DNA turns apart. Three potential binding sites were detected. Binding site 1 is about 3 kb upstream of the transcriptional start site (Figure 4C) and binding site 2 is around 1 kb upstream of the transcriptional start site (Figure 4C); binding site 3 is about 500 bp downstream of the transcriptional start site in the first intron (Figure 4C).

A 3.6-kb *exdpf* promoter region containing the transcriptional start and three potential PTF1-binding sites increased the activity of the luciferase reporter plasmid 41-fold compared to the promoterless pGL3-Basic in HEK 293 cell lines tested by transfection (Figure 4D, seq1). While deletion of binding site 1 reduced the activity to 29-fold, a 1.5 kb promoter region that retained binding site 2 and binding site 3 still maintained an activity of 26-fold (Figure 4D, seq 2 and seq 3). However, deletion of binding site 1 and binding site 3 reduced the activity to 8-fold (Figure 4D, seq4). Not surprisingly, deletion of all three binding sites further reduced the transcription of the reporter gene almost to a basal level (Figure 4D, seq 5). These results provide strong evidence that Ptf1a can promote the transcription of *exdpf*, and Ptf1-binding site 1 and binding site 3 are especially critical for the activation of the promoter. Taken together, these data demonstrate that *exdpf* is a direct target gene of Ptf1a.

### Excess *exdpf* Causes Increases in Exocrine Pancreas Mass

**Figure 5:**
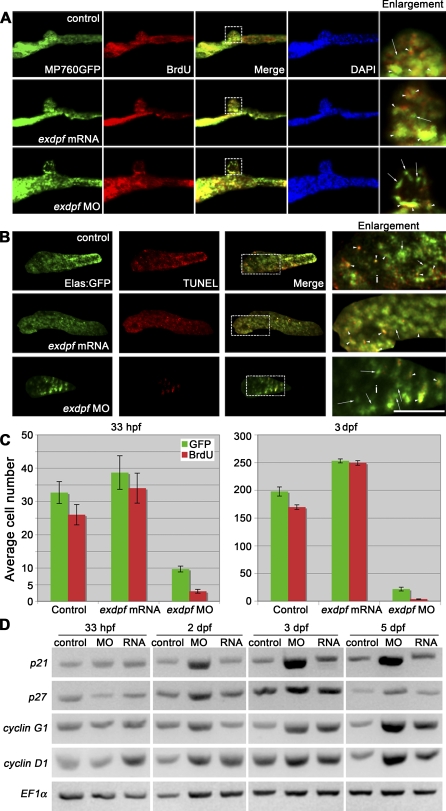
Reduced *exdpf* Causes Defects of Exocrine Cell Proliferation (A) BrdU labeling result in 33 hpf embryos. Green: MP760*:GFP*, Red: BrdU staining. Blue: DAPI staining. Dorsal view, anterior to the left. Enlargement represents higher magnification of boxed area in each row. Arrows indicate non-proliferating cells; arrowheads indicate proliferating cells as evidenced by overlapping of red and green colors. (B) BrdU labeling result in 3 dpf embryos. Green: *elastase A:GFP*, Red: BrdU staining. Lateral view, anterior to the left. Scale bar: 50 μm. Arrows indicate non-proliferating cells; arrowheads indicate proliferating cells as evidenced by overlapping of red and green colors. (C) Quantitative graphs for BrdU incorporation rate in 33 hpf or 3 dpf embryos. The average number of GFP positive cells with BrdU incorporation was obtained by counting BrdU-labeled GFP positive cells from five embryos. Y axis: Mean ± SD. (D) Semiquantitative RT-PCR examination of expression of *cyclin D1* and cell cycle inhibitors *p21*, *p27* and *cyclin G1* in control (control), *exdpf* morphants (MO), and *exdpf* mRNA injected embryos (RNA) at 33 hpf, 2 dpf, 3 dpf, and 5 dpf. In each group, 30 embryos were used to extract total RNA for RT-PCR.

To assess whether *exdpf* is sufficient for exocrine specification, we carried out overexpression experiments by injecting synthetic *exdpf* mRNA into embryos at the one-cell stage (Figure 5). *Elastase A:GFP* transgenic fish were used to facilitate the identification of exocrine cells. In this fish, GFP expression is controlled by the *elastase A* (*elaA*) regulatory sequence which allows exocrine specific GFP expression in larvae and adult [43]. At 3 dpf, the average exocrine cell number in the control embryo is 197.8 ± 8.2 (Figure 5C; Table 2, control, mean ± standard deviation [SD], *n* = 5). Overexpression of *exdpf* caused a mild increase of exocrine mass by about 28% (Figure 5C, Table 2, 253 ± 4.2). Conversely, knocking down *exdpf* by morpholino remarkably reduced exocrine cell number to about 10% of control level (21.6 ± 3.4) at 3 dpf (Figure 5C, Table 2). At 5 dpf, exocrine cell number was further increased by 42.8% in *exdpf* mRNA injected embryos (Figure S6D, 376.2 ± 6.3 cells per embryo) compared with that of the control embryo (Figure S6A, 263.4 ± 4.4 cells per embryo, *n* = 5). Interestingly, exocrine cell number in the *exdpf* morphants did not change much from 3 dpf to 5 dpf (from 21.6 ± 3.4 to 22.8 ± 3.8), indicating that the proliferation rate of the exocrine cells is affected in morphants.

**Table 2:**
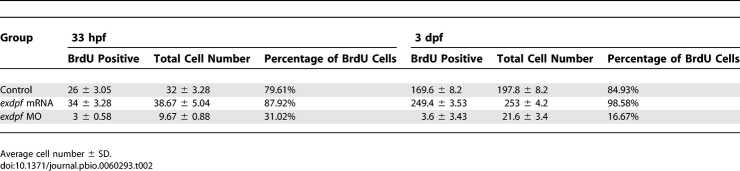
Quantitative Data of BrdU Assay

### Knocking Down *exdpf* Causes Cell Cycle Arrest but Not Apoptosis of the Exocrine Cells

Knocking down *exdpf* gene by antisense morpholino leads to loss or minimal formation of the exocrine pancreas. The strong reduction of the exocrine pancreas could be a result of increased cell death or decreased cell proliferation rate or a combination of both. To address these possibilities, we performed BrdU incorporation experiments to assess cell proliferation. We chose the transgenic line MP760GFP to assess exocrine pancreatic progenitor cell proliferation since GFP expression is observed in the pancreatic area at 33 hpf, 1 h before exocrine cell differentiation (Song J, unpublished data). At this stage, about 80% of exocrine pancreatic progenitors were proliferating in the control embryos injected with control morpholino (Figure 5A [top] and 5C, Table 2). Injection of *exdpf* mRNA increased GFP positive cell number by about 20% and 88% of the cells were proliferating (Figure 5A [center] and 5C, Table 2). In contrast, knocking down *exdpf* by morpholino caused a severe reduction of the exocrine progenitor cell number by 70% (Figure 5C, Table 2) and only 31% of the cells were proliferating (Figure 5A [bottom] and 5C, Table 2). These results indicate that *exdpf* is required for exocrine progenitor specification and proliferation.

For differentiated exocrine cells, *elastase A:GFP* transgenic fish was used to facilitate the identification of exocrine cells. We chose 3 dpf embryos to compare cell proliferating ability because exocrine cells are still undergoing massive proliferation at this stage. At 3 dpf, in the control embryos, the BrdU incorporation rate was about 85% (Figure 5B [top] and 5C, Table 2). Injection of *exdpf* mRNA increased the BrdU incorporation rate to about 98% (Figure 5B [center] and 5C, Table 2). This result suggests that *exdpf* is sufficient to promote exocrine cell proliferation. In contrast, knocking down *exdpf* by morpholino significantly reduced the BrdU incorporation rate to about 17% (Figure 5B [bottom] and 5C, Table 2), indicating that *exdpf* is also necessary for proliferation of differentiated exocrine cells.

To assess whether cell death could contribute to the severe reduction of exocrine cells in the *exdpf* morphants, we performed TUNEL assay on 5 dpf embryos because exocrine cell number seems unchanged much from 3 dpf to 5 dpf. In the control embryos, a small fraction of exocrine cells were undergoing apoptosis (Figure S6C [enlargement] and S6J, 6.65% ± 2.66%, *n* = 5). Overexpression of *exdpf* by injecting synthetic mRNA increased exocrine cell number by ∼40%. Only basal level of cell death was detected in these embryos (Figure S6F and S6J, 6.03% ± 2.43%, *n* = 5). Similarly, knocking down *exdpf* did not significantly increase the ratio of cells undergoing apoptosis (Figure S6I enlargement and S6J, 7.38% ± 3.24%, *n* = 5). This result indicates that reduced *exdpf* function did not cause massive cell death of the exocrine pancreas.

### *exdpf* Regulates Expression of Cell Cycle Genes

To address the molecular mechanisms causing cell cycle arrest in the *exdpf* morphants, semi quantitative RT-PCR was performed to examine the expression levels of cell cycle inhibitors *cyclin G1*, *p21^Cip^*, and *p27^Kip^*, as well as G1 to S phase regulator *cyclin D1*, at different developmental stages, including 33 hpf (just before exocrine cell differentiation), 2 dpf (exocrine cells surround the endocrine islet), 3 dpf (exocrine cells expand posteriorly), and 5 dpf (exocrine cells form nice pancreatic shape with head, trunk and tail). At 33 hpf, no discernible difference of either *p21^Cip^* or *p27^Kip^* or *cyclin G1* expression was detected in control, *exdpf* morphants, and *exdpf* mRNA injected embryos (Figure 5D). From 2 dpf to 5 dpf, a dramatic increase of *p21^Cip^* expression (Figure 5D) was observed in the *exdpf* morphants, whereas the expression level remained comparable in the control and *exdpf* mRNA injected embryos. Similarly, a significant increase of *p27^Kip^* expression was also observed in the *exdpf* morphants, although not as much as *p21^Cip^* expression (Figure 5D, second row). In addition, the expression of *cyclin G1* also slowly increased in the *exdpf* morphants from 2 dpf to 5 dpf. Taken together, our results suggest that knocking down *exdpf* leads to cell cycle arrests through up-regulation of *p21^Cip^*, *p27^Kip^*, and *cyclin G1*.

We have shown in our previous result that overexpression of *exdpf* increased exocrine cell number due to overproliferation of these cells (Figure 5B, [center]). We then confirmed this result by checking the expression level of *cyclin D1* through semiquantitative RT-PCR. At 33 hpf (just before exocrine cell differentiation), *cyclin D1* expression was slightly increased in the *exdpf* mRNA injected embryos (Figure 5D). From 2 dpf to 5 dpf, the increase of *cyclin D1* expression in the *exdpf* mRNA injected embryos became stronger. Interestingly, we also detected elevated expression of *cyclin D1* in the *exdpf* morphants from 2 dpf to 5 dpf. We postulate that this type of increase may be caused by *exdpf* independent mechanisms that control organ size. Due to the strong increase of cell cycle inhibitors in *exdpf* morphants, an increased level of *cyclin D1* is not enough to drive the cell into the proliferating phase. The semi-quantitative RT-PCR results are further confirmed by real-time PCR (Table 3). Similar up-regulation of cell cycle inhibitors including *p21*, *p27*, and *cyclin G1* were detected in the *exdpf* morphants (Table 3).

**Table 3:**
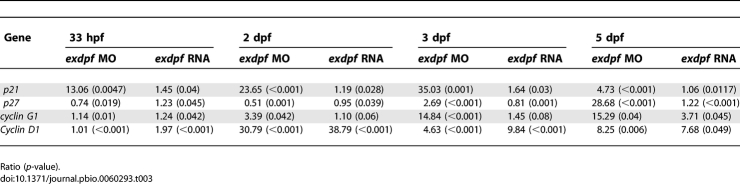
Real-Time PCR Analysis Of Cell Cycle Control Genes

To examine where the increase of *p21* expression comes from, we performed in situ hybridization using a *p21* probe. At 33 hpf, *p21* expression was detected in the developing brain (Figure S7A and enlargement). No *p21* expression was observed in the developing pancreatic area. A similar *p21* expression pattern was observed in *expdf* mRNA injected embryos (Figure S7B and enlargement). Conversely, noticeable *p21* expression could be seen in the developing pancreatic area in *exdpf* morphants (Figure S7C and enlargement). Thus, cell proliferation defects observed in the developing exocrine pancreas caused by knocking down *exdpf* are likely mediated by increased level of *p21* expression in the cells.

### The *exdpf* Gene Acts Genetically Downstream of RA in Regulating Exocrine Pancreas Development

**Figure 6:**
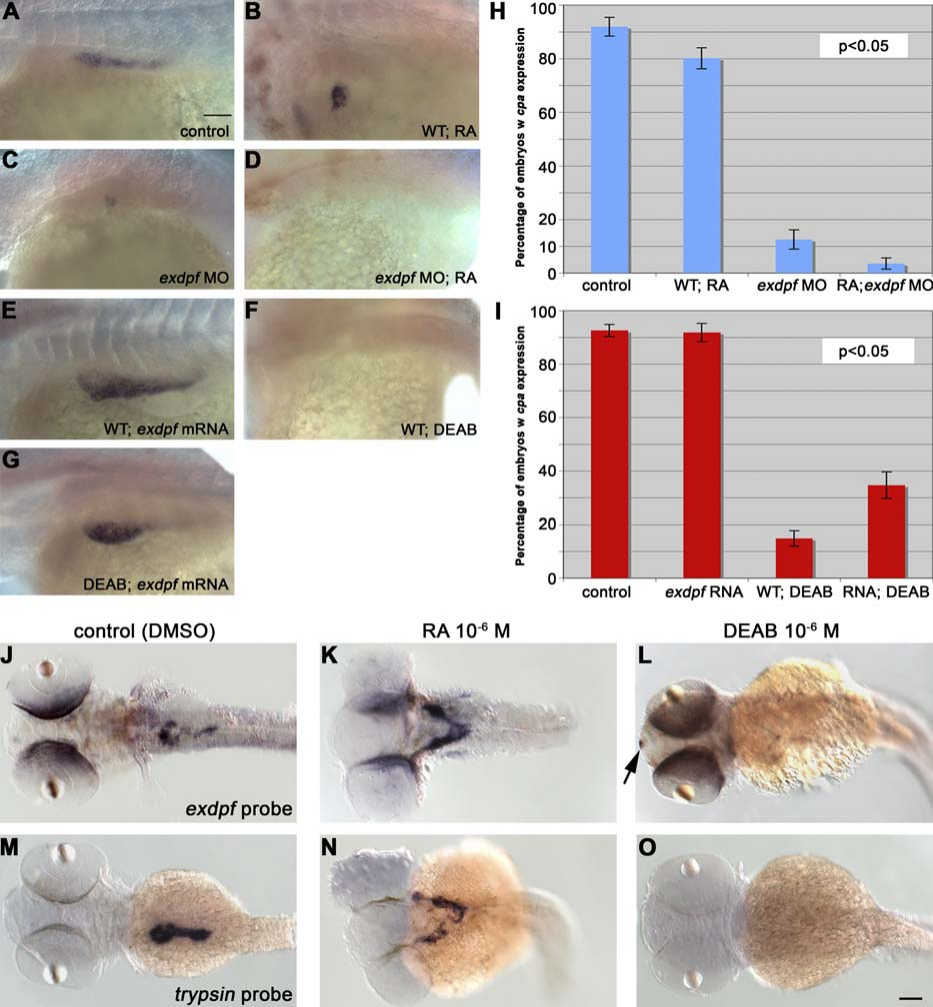
The *exdpf* Gene Acts Genetically Downstream of RA during Exocrine Pancreas Development (A–G) In situ hybridization on 3 dpf embryos using a *carboxypeptidase A* (*cpa*) probe. (A) A WT control embryo. Note posterior expansion of exocrine pancreas. (B) An example of WT embryo treated with 10^−6^ M RA. Note exocrine pancreas is restricted to the anterior part in the endogenous position. (C) An example of WT embryo injected with 2 ng of *exdpf* morpholino. Note exocrine pancreas is restricted to the head area in a similar fashion to (B). (D) An example of WT embryo injected with 2 ng of *exdpf* morpholino followed by treatment of 10^−6^ M RA. Note no *cpa* expression was detected. (E) A WT embryo injected with 100 pg of *exdpf* mRNA. Note the increase of exocrine pancreas. (F) A WT embryo treated with 10^−6^ M of DEAB. No *cpa* expression was detected. (G) An example of WT embryo injected with 100 pg of *exdpf* mRNA followed by treatment with 10^−6^ M of DEAB. Note *cpa* expression was detected but not fully expanded posteriorly. All embryos were mounted anterior to the left, dorsal-lateral view. Scale bar: 50 μm. (H) A quantitative graph showing the percentage of embryos with *cpa* expression corresponding to (A–D). Y-axis, percentage of embryos with *cpa* expression. Mean ± SD. (I) A quantitative graph showing the percentage of embryos with *cpa* expression corresponding to (E–G) and control (A). Y-axis, percentage of embryos with *cpa* expression. Mean ± SD. In (H, I), percentage numbers are obtained from three independent experiments (*n* = 3). P value is from two-tail *t*-test for two samples assuming unequal variance (experimental group versus control). (J–O) In situ hybridization results using an *exdpf* or *trypsin* probe. (J and M) WT embryos treated with DMSO as controls. (K and N) WT embryos treated with 10^−6^ M of RA. Note no *exdpf* or *trypsin* expression posterior to the white arrows. (L and O) WT embryos treated with 10^−6^ M of DEAB.

We have shown that *exdpf* is essential for the exocrine pancreas differentiation and expansion; overexpression of *exdpf* gene leads to increased exocrine size. This effect is similar to that of RA treatment (Figure 6). Embryos treated with RA exhibited expanded endocrine pancreas [25]. Exogenous RA treatment also caused ectopic formation of exocrine at positions anterior to the presumptive pancreatic area (Figure 6B). But the exocrine pancreas failed to expand toward the posterior at 5 dpf (unpublished data; only a small number of embryos survived) as it did in untreated control embryos. Interestingly, RA treatment of *exdpf* morphants failed to induce exocrine formation (Figure 6D). This result indicates that exocrine pancreas formation in RA-treated embryos requires *exdpf* function.

In reciprocal experiments, treatment with 10^−6^ M diethylaminobenzaldehyde (DEAB) to block the RA pathway often resulted in no exocrine pancreas formation at 3dpf (Figure 6F, 85%, *n* = 181 embryos). Only about 15% (*n* = 181) of DEAB treated embryos exhibited weak expression of an exocrine marker at 3 dpf. This result suggests that RA is required for early exocrine formation during normal pancreas development. To test whether overexpression of *exdpf* can rescue exocrine pancreas formation in the DEAB treated embryos, we injected synthetic *exdpf* mRNA into embryos at the 1-cell stage followed by treatment with 10^−6^ M of DEAB from 9 hpf up to the time of analysis. At 3 dpf, *exdpf* mRNA injection increased the percentage of embryos exhibiting expression of the exocrine marker *cpa* to 35% (Figure 6G and 6I, *n* = 166). This result suggests that *exdpf* acts downstream of the RA pathway during normal pancreas development.

RA treatment often resulted in bilateral ectopic exocrine formation at the anterior area (Figure 6N, 83%, *n* = 191 embryos). To test whether exogenous RA treatment induces *exdpf* expression, in situ hybridization using an *exdpf* probe was performed. Interestingly, similar bilateral ectopic expression of *exdpf* was observed in RA treated embryos (Figure 6K). On the other hand, blocking RA synthesis by DEAB completely abolished *exdpf* expression in the pancreatic area (Figure 6L), whereas the epiphysis expression appeared normal (Figure 6L, arrowhead). These data suggest RA signaling influences *exdpf* expression in the developing pancreas.

Taken together, these results place *exdpf* genetically downstream of RA in exocrine pancreas development.

### Overexpression of *exdpf* Inhibits Endocrine β Cell Development

**Figure 7:**
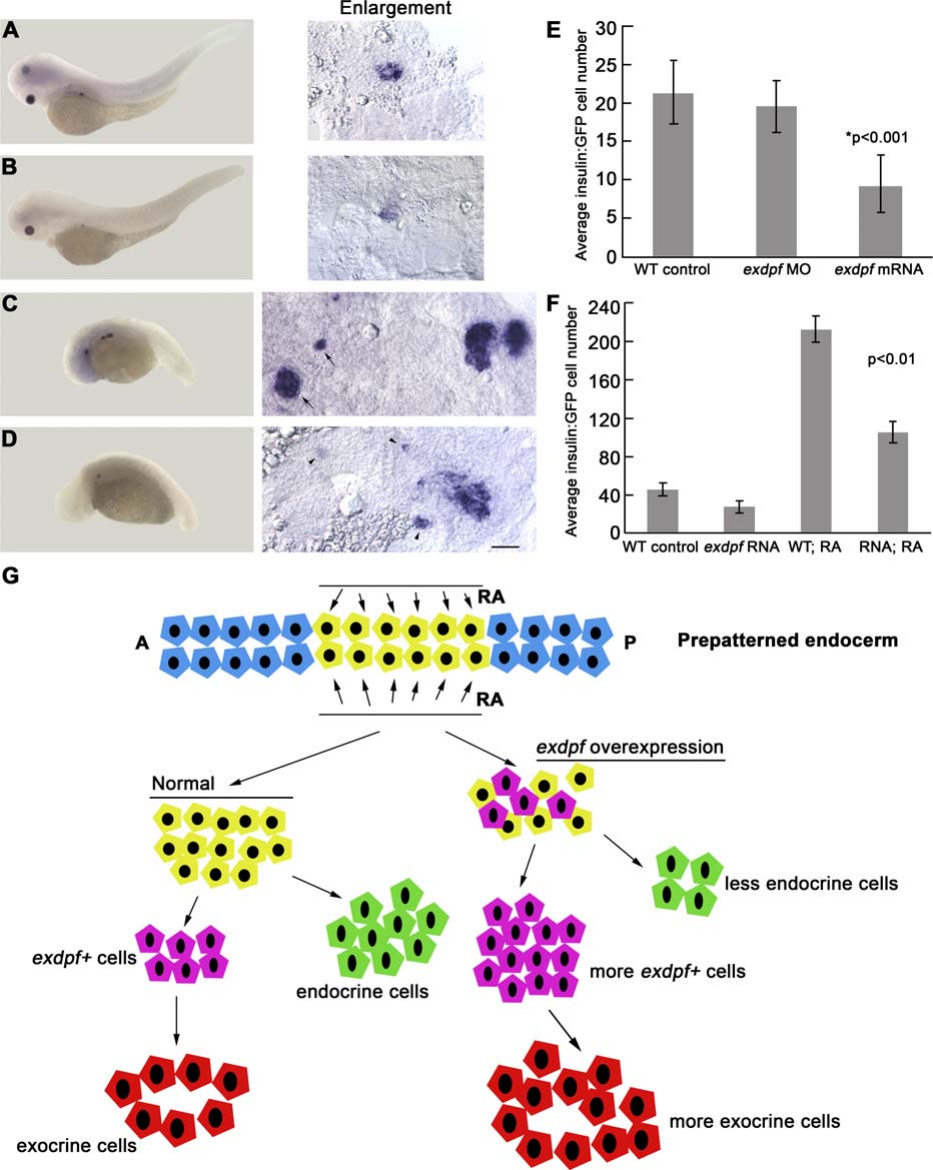
Overexpression of *exdpf* Represses Endocrine Cell Differentiation in Both WT and RA-Treated embryos (A–D) In situ hybridization on 3 dpf embryos using a *preproinsulin* probe. Enlargement: higher magnification of pancreatic area. (A) A WT embryo treated with DMSO as control. (B) A WT embryo injected with 100 pg of *exdpf* mRNA. (C) A WT embryo treated with 10^−6^ M of RA. Note anterior expansion of endocrine cells at the endogenous position and anterior ectopic endocrine cells (arrows). (D) A WT embryo injected with 100 pg of *exdpf* mRNA followed by RA treatment. Note weak anterior ectopic endocrine cells (arrowheads). Scale bar: 50 μm. (E) A quantitative graph showing average *GFP*-positive cells per *preproinsulin:GFP* embryo at 24 hpf. Y axis, Mean ± SD. (F) A quantitative graph showing average *GFP*-positive cells per *preproinsulin:GFP* embryo at 3 dpf. Y axis, Mean ± SD. (G) A model for the effect of *exdpf* on endocrine cell differentiation. Normally, *exdpf* is only expressed in the exocrine cell precursors after endocrine cell differentiation. Injection of *exdpf* mRNA caused ectopic expression of *exdpf* in the committed pancreatic progenitors before endocrine cell differentiation. Therefore, more progenitors are transformed into exocrine cell fate and fewer progenitors develop into endocrine cells.

The expression of the *exdpf* gene is excluded from the endocrine islet during pancreatic development (Figure 1I). To evaluate the effect of *exdpf* overexpression on endocrine cell differentiation, we performed in situ hybridization against *preproinsulin* in 3 dpf embryos (Figure 7). In the *exdpf* mRNA injected embryos, *preproinsulin* expression was dramatically reduced (Figure 7B). *Preproinsulin:GFP* transgenic fish were injected with *exdpf* mRNA to quantify the GFP-positive cells. In the control embryos, the average number of GFP positive cells was 45.15 ± 7.04 (Figure 7F, mean ± SD, *n* = 20). As expected, overexpression of *exdpf* resulted in a significant reduction of GFP positive cell number by about 40%; the average number of GFP-expressing cell was 27.55 ± 6.02 (Figure 7F, *n* = 20). The stage of 3 dpf is relatively late for β cell development. To examine whether the reduction of β cell number at 3 dpf is due to defects in cell proliferation or specification, we quantified *preproinsulin:GFP* positive cells at 24 hpf (see Figure S8 for panel of embryos used for quantification) when the majority of β cells come from newly specified cells. Overexpression of *exdpf* reduced β cell number by about 43% (Figure 7E, from 21.4 ± 4.16 to 9.2 ± 4.04 cells per embryo). However, no significant change in β cell number was observed in the *exdpf* morphants (Figure 7E, 19.5 ± 3.39 cells per embryo). These data indicate that overexpression of *exdpf* inhibits β cell specification and suggests a possible transformation of cell fate in the endocrine pancreatic precursors.

Exogenous RA treatment of WT embryos dramatically increased *preproinsulin*-expressing cell number (Figure 7C and 7F, 212.15 ± 14.88 cells per embryo, *n* = 20) compared with that in the DMSO treated control embryos (Figure 7A and 7F, 45.15 ± 7.04, *n* = 20). Interestingly, overexpression of *exdpf* in the RA treated embryos inhibited the anterior expansion of the endocrine pancreas (Figure 7D and 7F, 105.2 ± 10.88 cells per embryo, *n* = 20). This result suggests that overexpression of *exdpf* in the anterior ectopic pancreas induced by RA treatment balanced RA signaling and turned more cells into the exocrine fate.

## Discussion

Our results provide strong evidence that a novel gene *exdpf* is specifically employed by the zebrafish exocrine progenitors to promote cell differentiation and proliferation. Moreover, epistasis experiments support that *exdpf* functions downstream of *ptf1a* in exocrine cell specification. RA also interacts genetically with Exdpf during exocrine formation.

How does Exdpf regulate exocrine cell specification? First, *exdpf* starts to express in exocrine progenitors at 33 hpf, just before exocrine cell differentiation. Second, *exdpf* is required for exocrine cell differentiation. Knocking down *exdpf* by antisense morpholino resulted in the absence of exocrine markers in a majority of injected embryos. The remaining smaller fraction of *exdpf* morphants exhibited significantly reduced expression of exocrine markers. Third, *exdpf* is both necessary and sufficient for exocrine cell proliferation. We observed *exdpf* overexpression increased the proliferation of exocrine cells (98% versus 85% in the control embryos), whereas knocking down *exdpf* severely impaired proliferating ability due to the increased level of cell cycle inhibitors *p21^Cip^*, *p27^Kip^*, and *cyclin G1*. Although *exdpf* is necessary for exocrine cell differentiation, it is not sufficient to induce ectopic formation of the exocrine pancreas. A possible explanation is that *exdpf* only functions in the committed exocrine progenitors. Our result shows that Exdpf promotes exocrine cell proliferation by regulating *cyclin D* expression.

Exdpf acts genetically downstream of *ptf1a* in exocrine specification. It is likely that *ptf1a* controls the expression of *exdpf* in exocrine progenitors. The expression pattern of *exdpf* in exocrine pancreas is similar to that of *ptf1a* [36]. *ptf1a* is expressed in the exocrine progenitors at 32 hpf. Similarly, *ptf1a*-expressing cells also surround β cells as a result of gut rotation [36]. In addition, three Ptf1a binding sites have been identified in the promoter region of the *exdpf* gene (Figure 4C). Using luciferase assay, we were able to show that these Ptf1a binding sites are functional in culture cells. Thus, *exdpf* is a direct target gene of Ptf1a. Other transcription factors such as Pdx1 might also control exocrine progenitor specific expression of *exdpf*. During embryogenesis, *ptf1a* is expressed in the ventral aspect of *pdx1*-positive domain from 32 hpf to 36 hpf [36]. Moreover, two Pdx1 binding sites have also been identified within *exdpf* promoter region (data not shown). Knocking down of *ptf1a* leads to the agenesis of the exocrine pancreas, which is consistent with previous results [36,37]. Injection of *exdpf* mRNA into the *ptf1a* morphants successfully restored the expression of the exocrine marker. This is likely due to the expansion and differentiation of residual progenitor cells promoted by *exdpf* mRNA in the *Ptf1a* morphants. In our rescue experiments, the injected concentration of *ptf1a* morpholino only created a hypomorph situation and should still contain a residual pool of progenitors. Co-injection of *exdpf* mRNA promotes proliferation and differentiation of these cells, resulting rescue of the defect. It is also likely that Ptf1a activity recovers following cessation of the morpholino effect, and that this allows for full exocrine differentiation to occur in progenitor cells that have been rescued by exogenous *exdpf*. Nonetheless, our results provide evidence that Exdpf acts downstream of *ptf1a* in exocrine formation. In addition, overexpression of *exdpf* by mRNA injection inhibited endocrine cell fates (Figure 7B, 7E, and 7F). This endocrine repression result is in agreement with a recent report by Dong et al. Using partial loss of function analysis for *ptf1a*, Dong et al. found that high levels of *ptf1a* promote exocrine fate whereas low levels promote endocrine fate [44]. It is likely that *ptf1a* exerts its function through *exdpf* in this aspect.

The *exdpf* gene encodes a putative signaling molecule containing two SH2 and two SH3 domains as well as other conserved domains (Figure S4), suggesting that Exdpf may function in response to signals from adjacent mesoderm tissues. Multiple intercellular signals including transforming growth factor beta, Hedgehog, and Notch are critical for the proper specification of endocrine and exocrine cell fates during pancreas development. It is not clear which signal or signals *exdpf* responds to in order to make the exocrine cell fate decision. Our results support the idea that *exdpf* promotes exocrine cell proliferation. As a putative signaling molecule, *exdpf* might be involved in transducing signals from growth factors that are required for exocrine cell proliferation. However, the identities of such growth factors remain elusive.

Genetic evidence places *exdpf* downstream of RA. Exogenous treatment with RA caused anterior expansion of endocrine cells as well as ectopic anterior exocrine cells, which is consistent with previous results [25]. Contradictory results from RA treatment have been reported using different organisms regarding exocrine development. In mouse embryonic culture, RA treatment suppresses exocrine differentiation and branching morphogenesis [30,45]. Others reported that *at*RA treatment leads to endocrine and duct differentiation from the pancreatic bud, but inhibits exocrine differentiation [45]. In *Xenopus*, exogenous RA treatment causes endocrine expansion in the dorsal bud at the expense of exocrine tissue but stimulates exocrine differentiation in the ventral bud [26]. These contradictory results might derive from different organisms and different concentrations of either *at*RA or 9cis RA. In our experiment, we find that excessive *at*RA caused ectopic anterior exocrine formation. Exogenous treatment with RA induced ectopic pancreatic cells including endocrine and exocrine cells. Anterior ectopic formation of the exocrine pancreas by RA treatment requires *exdpf* function since *exdpf* morpholino injection blocks ectopic exocrine formation and RA also induces anterior ectopic expression of *exdpf*. Overexpression of *exdpf* in wild-type embryos significantly suppresses endocrine cell differentiation, suggesting *exdpf* transforms the cell fate of pancreatic progenitors (Figure 7G). The balance of RA and overexpressed *exdpf* in the progenitors results in reduced endocrine cells compared with RA treated wild-type embryos.

Pancreatic cancer is one of the leading causes of cancer deaths because it is often highly aggressive and resistant to treatments available at the time of diagnosis [46]. Genetic studies have identified structural mutations in pancreatic cancers; the alterations include the activation of *K-Ras* proto-oncogene as well as inactivation of tumor suppressor genes such as *TP53* or *INK4a* locus [47–49]. By carefully searching the NCBI database, we found that the human *exdpf* ortholog is expressed in relatively high levels in multiple tissues including the pancreas, colon, and mammary glands. Interestingly, the EST expression profile also indicates that higher level of *exdpf* ortholog has been detected in several cancers including pancreatic cancer, breast cancer and kidney cancer (Figure S9). In addition to structural mutations, many growth factor receptors and their ligands are overexpressed in pancreatic cancers. Since *exdpf* can promote acinar cell proliferation, it is worth studying the role of this gene in pancreatic cancer. Zebrafish has proven to be a useful model system to study pancreatic cancers. In 2004, Yang et al. reported that human MYCN caused pancreatic neuroendocrine tumors in transgenic zebrafish that expressed MYCN in β cells, muscle cells and neurons [50]. Recently, transgenic fish that express oncogenic KRAS^G12V^ under the *ptf1a* promoter was generated. In these fish, KRAS^G12V^ blocked the differentiation of pancreatic progenitor cells and this undifferentiated progenitor pool lead to invasive pancreatic cancer [51]. These examples demonstrate that the zebrafish model is useful in advancing our understanding of pancreatic cancers.

Together, our results reveal a specific requirement for Exdpf in exocrine pancreas formation in zebrafish. The gene *exdpf* is expressed exclusively in the exocrine progenitors and differentiated exocrine cells. We demonstrate that *exdpf* is necessary for exocrine cell differentiation. Furthermore, *exdpf* is both sufficient and necessary for the proliferation of differentiated exocrine cells. We speculate that the study of the function of *exdpf* in pancreatic cancers could shed light on the pathogenesis of this malignancy.

## Materials and Methods

### Zebrafish husbandry.

Zebrafish were raised and kept under standard laboratory conditions at about 28 °C. Embryos were staged according to Kimmel et al. [52]. The *elastase A:GFP* fish was a gift from Gong's laboratory in Singapore [43]. The wild-type line used was AB.

### In situ hybridization.

In situ hybridization was performed essentially as previously described [53]. For double in situ hybridization, Fast Red (Roche) and NBT/BCIP (50 mg/ml; Promega) were used as alkaline phosphatase substrates. The following probes were used: *preproinsulin* [54], *trypsin*, *cpa*, *ptf1a*, *p21*, and *exdpf*.

### Chemical treatment.

For RA treatment, wild-type zebrafish embryos were incubated with retinoic acid as described [25]. For DEAB treatment, embryos were incubated with 10^−6^ M DEAB diluted from 10^−2^ M stock solution in DMSO as described [55].

### RNA synthesis and injection.

mRNAs of *exdpf* and *ptf1a* were synthesized using T7 or SP6 mMessage mMachine kit (Ambion). mRNA injection was performed as described at the one-cell stage [56]. Sterile water was used for the control experiments. 100 pg of *exdpf* or *ptf1a* mRNA was used for all experiments.

### Injection of antisense morpholino oligonucleotides.

Antisense morpholino oligos (MOs) designed against *exdpf*: MO1: 5′-GCTGGATGGAATTGCTGCCATTTTC-3′. MO2: 5′-TCGACCGTGTGGAAGATGGAAAGAT-3′ were obtained from Gene Tools. *Ptf1a*-MOa, 5′-AGTGTCCATTTTTTGTGCTGTGTTG-3′ was obtained from Open Biosystems and injected into one- to two-cell stage embryos (for original reference, see [36]). A morpholino standard control oligo (5′-CCTCTTACCTCAGTTACAATTTATA-3′) used as control was obtained from Gene Tools. All morpholinos were prepared and resuspended in 1× Danieau's buffer at 1 ng/nl for injection.

### BrdU staining.

Embryos were placed in 10 mM solution of BrdU (Sigma) in fish water at 24 hpf and kept in dark at 28 °C for 48 h. Then embryos were fixed in 4% PFA for 2 h at room temperature. After washing (5 × 5 min in PBST), embryos were incubated in 1 N HCl for 1 h. Then embryos were washed again for 5 × 5 min in PBST and blocked using 5% goat serum for 1 h at room temperature and incubated over night at 4 °C in 1:100 mouse anti-BrdU monoclonal antibody (Chemicon) and 1:500 rabbit anti-GFP polyclonal antibody (Abcam). After washing (5 × 5 min in PBST), embryos were incubated overnight at 4 °C in 1:200 A488 conjugated goat anti-rabbit antibody (Molecular Probe). Embryos were then washed and incubated in 1:200 TRITC conjugated rabbit anti-mouse antibody (Sigma) over night at 4 °C. The stained embryos were incubated with DAPI for 30 min and visualized via a Zeiss LSM 510 Laser Confocal Microscope.

### TUNEL assay.

The terminal deoxynucleotidyl transferase–mediated dUTP-fluoroscein nick-end labeling (TUNEL) assay was performed essentially as described previously [57]. Embryos were fixed overnight in 4% PFA in PBS at 4 °C and permeabilized using methanol. Then embryos were rehydrated and washed 5 × 5 min in PBST at room temperature. TUNEL labeling was performed by 1 h incubation at 37 °C in a cell death detection reagent (in situ cell death Detection Kit-TMR Red, Roche Diagnostics). As negative controls, embryos were incubated in the TUNEL label only. For positive controls, embryos were treated with DNaseI for 1 h at 37 °C before TUNEL labeling. After the reaction, embryos were washed 4 × 15 min in PBST at room temperature and stored in PBST at 4 °C in a covered canister. Fluorescence was detected using a Zeiss LSM 510 Laser Scanning Confocal Microscope.

### Imaging and quantification.

Images were captured using a digital camera (Axiocam) attached to a compound microscope (Zeiss: Axioplan 2 or Imager A1) and Improvision (an Openlab program) or Axiovision. Confocal images were captured using a Zeiss LSM 510 Laser Scanning Confocal Microscope. To quantify *elastase A:GFP*-positive cell numbers, yolk was removed from the embryo and the rest of the embryo was flattened by cover slide. GFP-positive cells were used to outline exocrine and DAPI staining inside the exocrine area was counted under a compound microscope. Calculations were performed using Microsoft Excel. We report mean and standard deviation of exocrine cell numbers. The probability associated with the Student's *t*-test (with two-tailed distribution) and two samples of unequal variance were also included.

### RT-PCR and real-time PCR.

RT-PCR was performed for transcript analysis of *p21^Cip^*, *p27^Kip^*, *cyclin D1*, *cyclin G1*, and *EF1α*. Total RNA was extracted from 30 embryos of each group at various developmental stages using Qiagen Rneasy Mini kit (Qiagen, Valencia, CA). The total RNA pellet was resuspended in 30 μl of RNase free water and stored at −80 °C. For reverse transcription, 10 μl of RNA was mixed with 1 μl of random primer and incubated for 5 min at 68 °C and placed on ice. For each reaction, a mix of 4 μl of 5× RT buffer (50 mM Tris-HCl, 75 mM KCl, 3 mM MgCl_2_; Invitrogen, Carlsbad, CA), 2 μl of dNTPmix (10 mM), 2 μl of DTT (100 mM), and 1 μl of M-MLV reverse transcriptase (200 U; Invitrogen, Carlsbad, CA, Canada) was added and samples were incubated for 1 h at 42 °C followed by 10 min at 68 °C. To test for genomic DNA contamination, equivalent RNA samples were treated in the same manner, except that RNase-free water was added to the reaction instead of the reverse transcriptase. RT-PCR primer sequences and reaction conditions are presented in Table S1. Real-time PCR was performed using Bio-Rad CYBR Green Supermix on iCycler. For each time point, triplicate was used to determine the ratio of experimental group versus control. Standard curve method was used. *p*-Values were obtained using the Student's *t*-test (with two-tailed distribution) and two samples of unequal variance.

### Luciferase assay.

*Exdpf* promoter sequences were generated by the polymerase chain reaction (PCR) with Long Taq PCR Mastermix (TIANGEN) from genomic DNA of AB zebrafish. The fragments of the *exdpf* promoter obtained were inserted into the pGL3-Basic vector (Promega).

Zebrafish *Ptf1a*, human *E47* and *RBP-J* expression constructs were kindly provided by Steven D. Leach [58], Michael Chin [59], and S. Diane Hayward [60], respectively. Human embryonic kidney 293 cells were plated at 70% confluence in 24-well plates and transfected with 0.8 μg of DNA per well using Lipofectamine 2000 (Invitrogen) following the manufacturer's protocol. A *Renilla* luciferase control plasmid (Promega Dual-Luciferase Reporter Assay System) was included in all transfections to allow normalization for transfection efficiency. Total DNA content per well was made consistent by supplementing with pCDNA3.1(+) vector when necessary. Ptf1-activated luciferase activity was defined as the ratio of firefly to *Renilla* luciferase luminescence and is further normalized against luciferase activity from cells transfected with the pGL3-Basic construct. All values are the means of at least three transfections ± standard error of the mean.

## Supporting Information

Figure S1 Vertebrate Orthologs of Exdpf are Highly Conserved (A) Peptide sequences of zebrafish Exdpf and Exdpfh (Endpf) aligned with mouse and human c20orf149 proteins using the ClustalW WWW Service at the European Bioinformatics Institute (http://www.ebi.ac.uk/clustalw; (Thompson et al., 1994). ‘*' indicate positions that have a single, fully conserved residue. ‘:' and ‘.' indicate positions that have strong (:) and weak (.) similarities. Dashes indicate gaps.
(B) Comparison of the genomic location of *exdpf* and *endpf* genes in zebrafish and human. Lines connecting locations indicate homologous genes.
(2.8 MB TIF)

Figure S2 RT-PCR Result of *exdpf* During Embryogenesis Note *exdpf* can be detected at one-cell stage, indicating that the transcripts are maternally deposited into the eggs. Zygotic expression of *exdpf* starts at around shield stage and increases during subsequent embryogenesis. A strong level of *exdpf* expression can be detected at 2 dpf and lasts until 5 dpf, the longest time point of this analysis.
(165 KB TIF)

Figure S3 *Exdpf* Antisense Morpholino Oligonucleotide MO2 Inhibits Exocrine Pancreas Formation (A and B) In situ hybridization using a *carboxypeptidase A (cpa)* probe. (A) A wild-type (WT) embryo injected with 2 ng of standard morpholino oligonucleotide control. White arrow head: *cpa* expression in pancreas. (B) An embryo injected with 2 ng of *exdpf* MO2. Note severely reduced expression of *cpa* (white arrow).
(C) An embryo injected with 4 ng of *exdpf* MO2.
(D) An embryo injected with 6 ng of *exdpf* MO2.
(1.2 MB TIF)

Figure S4 Predicted Domains of Exdpf Protein PROSITE (http://www.expasy.org/prosite) was used to predicate functional domains in Exdpf protein. The predicated domains and phosphorylation site as well as ATP binding site are listed.
(949 KB TIF)

Figure S5 *Exdpf* is Specifically Required for Exocrine Pancreas Formation but not Gut or Liver (A–C) Expression of GFP MP760GFP transgenic fish. (A) A control embryo injected with standard control morpholino. Note strong GFP expression in the pancreatic area at 24 hpf (arrow) and 2 dpf (arrowhead). (B) An example of embryo injected with *exdpf* mRNA (100 pg). Note strong GFP expression in the pancreatic area at 24 hpf (arrow) and 2 dpf (arrowhead). GFP expression in the developing gut is comparable to that in control embryo. (C) An example of embryo injected with *exdpf* morpholino (2ng of MO1*^exdpf^*). Note no strong GFP expression in the presumed pancreatic area at 24 hpf (arrow). A weak GFP positive pancreatic bud can be observed at 2 dpf (arrowhead). GFP expression in the gut is still comparable to that in the control and exdpf RNA injected embryos.
(D–G) In situ hybridization using a *ceruloplasmin* probe with 3 dpf embryos. (D) A control embryo injected with standard morpholino control. (E–G) Examples of embryos injected with *exdpf* morpholino. Note comparable expression of *cerulaplasmin* in all embryos.
(6.2 MB TIF)

Figure S6 Reduced *exdpf* Does not Affect Apoptosis in the Developing Pancreas (TUNEL assay result in 5 dpf embryos.
(A–C) A control embryo injected with standard morpholino control. Enlargement: of boxed area in (C). Note a few cell debris representing cells undergoing apoptosis (arrows in C enlargement).
(D–F) An embryo injected with 100 pg of *exdpf* mRNA. Enlargement: of boxed area in (F). Note comparable number of cells undergoing apoptosis (arrows).
(G–I) An embryo injected with *exdpf* morpholino. Enlargement: of boxed area in (I). Note both exocrine cells (arrows) and non-exocrine cells (arrowhead) undergoing apoptosis. Lateral view, anterior to the left. Scale bar: 50 μm.
(J) A quantitative graph of TUNEL assay.
(2 MB TIF)

Figure S7 Reduced *exdpf* Specifically Increases *p21* Expression in the Pancreatic Area In situ hybridization using a *p21* probe.
(A) A control embryo injected with standard morpholino control. Note *p21* expression in the head area. No *p21* expression is detected in the pancreatic area.
(B) An embryo injected with *exdpf* mRNA (100 pg). Note *p21* expression in the head area. No *p21* expression is detected in the pancreatic area.
(C) An embryo injected with *exdpf* morpholino. Note *p21* expression in the pancreatic area (arrow).
(3.5 MB TIF)

Figure S8 Examples of *preproinsulin:GFP* Embryos Used for Cell Counting Green: *preproinsulin:GFP*. Blue: DAPI staining. All embryos are at 24 hpf, de-yolked and flat mounted.
(6 MB TIF)

Figure S9 Expression of Human *exdpf* in Normal Tissues and in Tumors Human *exdpf* ortholog is expressed in different organs including colon, kidney, liver, and pancreas. Relatively higher level of *exdpf* has been detected in several tumors including colorectal caner, kidney tumor, liver tumor, and pancreatic tumor.
(741 KB TIF)

Table S1 Primers Used for RT-PCR and Real-Time PCR The sequences of forward and reverse primers and product length for each gene tested are included.
(27 KB DOC)

## Abbreviations

*at*RA: *all-trans* retinoic acid
Cpa: carboxypeptidase A
DEAB: diethylaminobenzaldehyde
dpf: day(s) postfertilization
E[number]: embryonic day [number]
Exdpf: exocrine differentiation and proliferation factor
GFP: green fluorescent protein
hpf: hour(s) postfertilization
Pdx1: pancreatic-duodenal homeobox 1
Ptf1a: pancreatic transcription factor 1, alpha subunit
RA: retinoic acid
RT-PCR: reverse transcriptase PCR
SD: standard deviation
