# Supplementary figures and images for: Exdpf Is a Key Regulator of Exocrine Pancreas Development Controlled by Retinoic Acid and ptf1a in Zebrafish
Source: PLoS Biol. 2008 Nov 25;6(11):e293. doi: 10.1371/journal.pbio.0060293 (PMC2586380; doi:10.1371/journal.pbio.0060293)

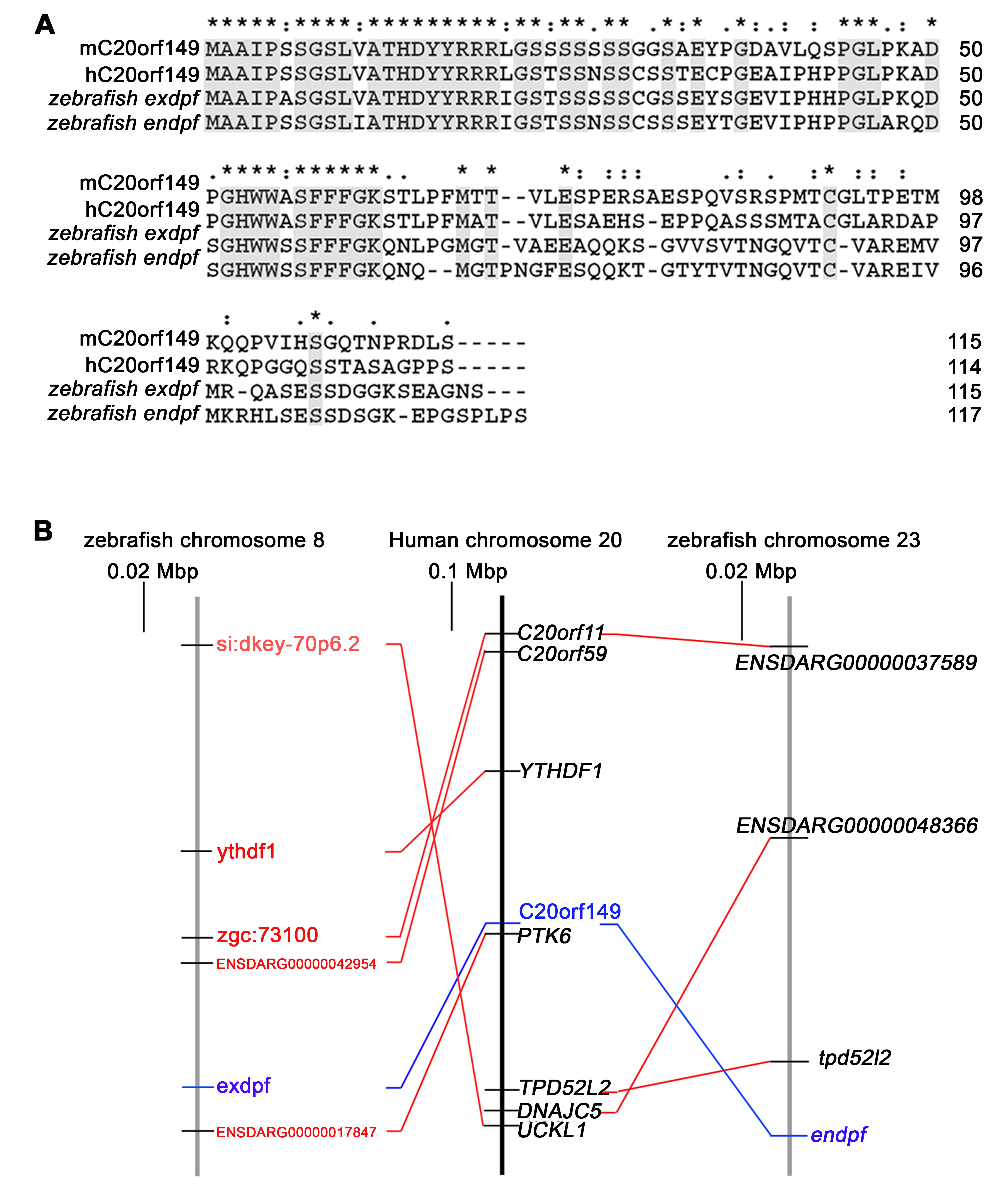

Supplement: Figure S1 — (A) Peptide sequences of zebrafish Exdpf and Exdpfh (Endpf) aligned with mouse and human c20orf149 proteins using the ClustalW WWW Service at the European Bioinformatics Institute (http://www.ebi.ac.uk/clustalw; (Thompson et al., 1994). ‘*' indicate positions that have a single, fully conserved residue. ‘:' and ‘.' indicate positions that have strong (:) and weak (.) similarities. Dashes indicate gaps. (B) Comparison of the genomic location of exdpf and endpf genes in zebrafish and human. Lines connecting locations indicate homologous genes. (2.8 MB TIF) [file pbio.0060293.sg001.tif]

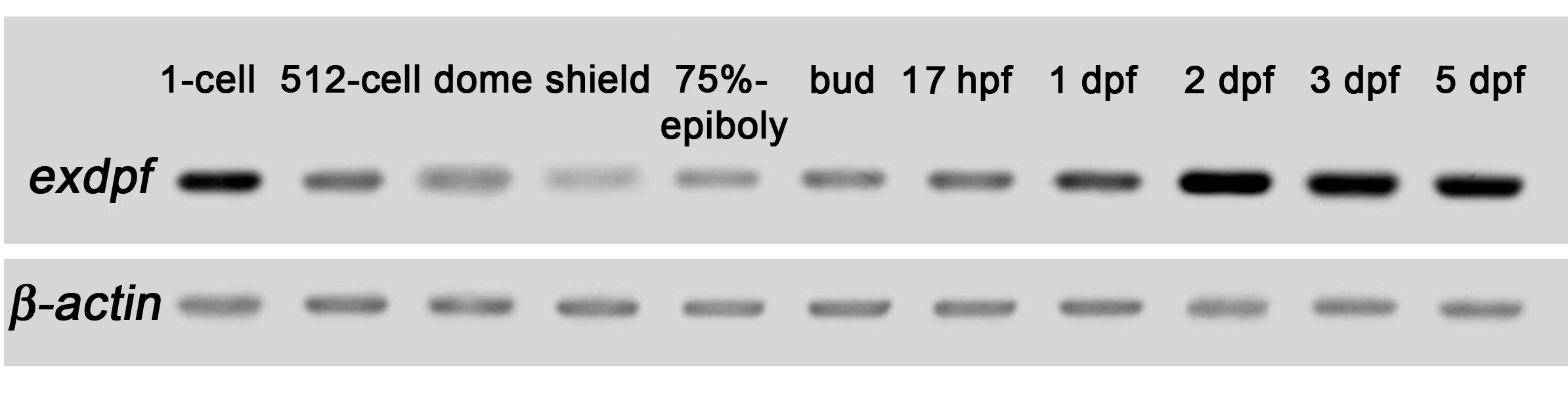

Supplement: Figure S2 — Note exdpf can be detected at one-cell stage, indicating that the transcripts are maternally deposited into the eggs. Zygotic expression of exdpf starts at around shield stage and increases during subsequent embryogenesis. A strong level of exdpf expression can be detected at 2 dpf and lasts until 5 dpf, the longest time point of this analysis. (165 KB TIF) [file pbio.0060293.sg002.tif]

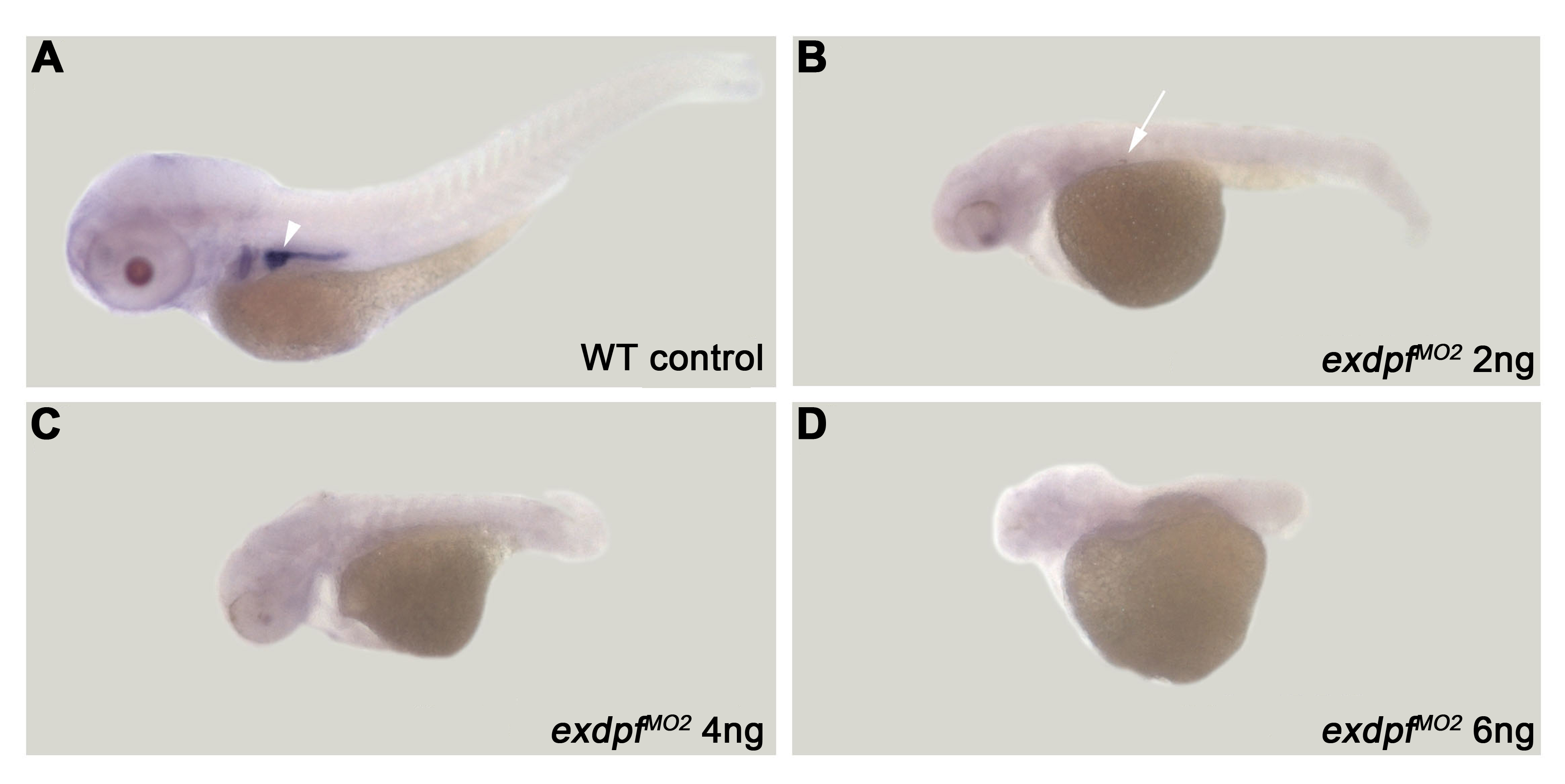

Supplement: Figure S3 — (A and B) In situ hybridization using a carboxypeptidase A (cpa) probe. (A) A wild-type (WT) embryo injected with 2 ng of standard morpholino oligonucleotide control. White arrow head: cpa expression in pancreas. (B) An embryo injected with 2 ng of exdpf MO2. Note severely reduced expression of cpa (white arrow). (C) An embryo injected with 4 ng of exdpf MO2. (D) An embryo injected with 6 ng of exdpf MO2. (1.2 MB TIF) [file pbio.0060293.sg003.tif]

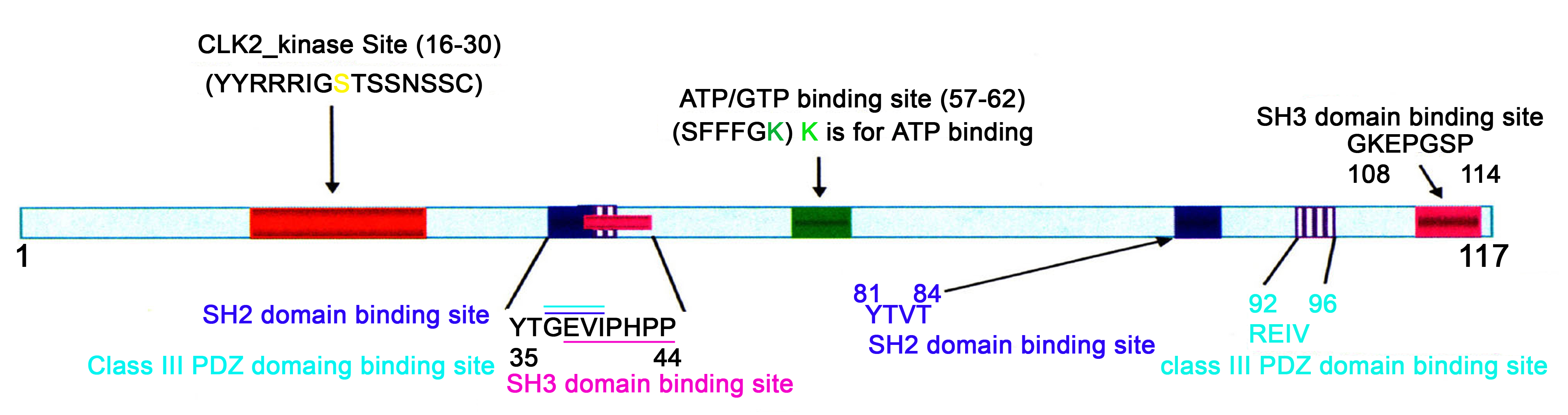

Supplement: Figure S4 — PROSITE (http://www.expasy.org/prosite) was used to predicate functional domains in Exdpf protein. The predicated domains and phosphorylation site as well as ATP binding site are listed. (949 KB TIF) [file pbio.0060293.sg004.tif]

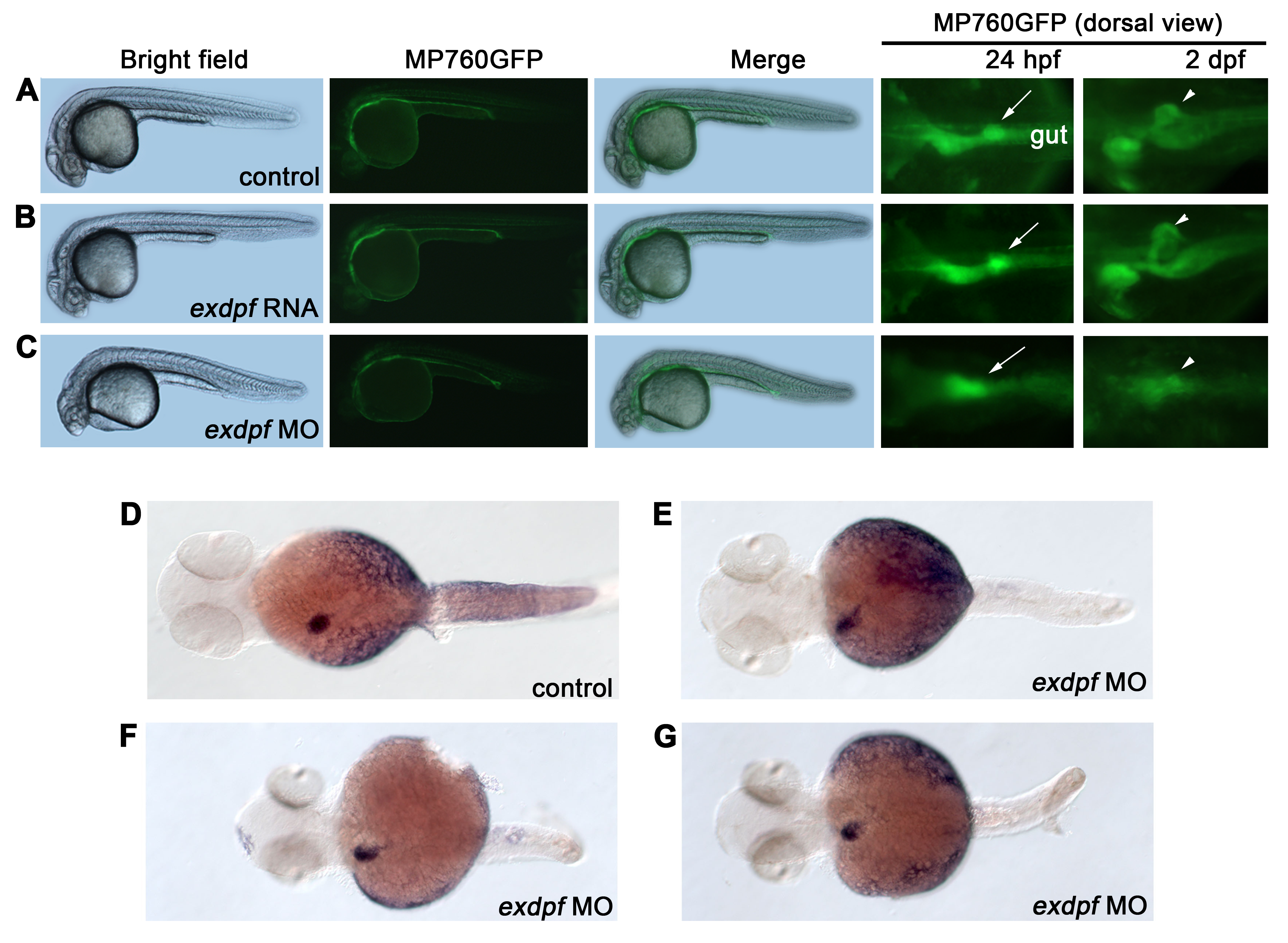

Supplement: Figure S5 — (A–C) Expression of GFP MP760GFP transgenic fish. (A) A control embryo injected with standard control morpholino. Note strong GFP expression in the pancreatic area at 24 hpf (arrow) and 2 dpf (arrowhead). (B) An example of embryo injected with exdpf mRNA (100 pg). Note strong GFP expression in the pancreatic area at 24 hpf (arrow) and 2 dpf (arrowhead). GFP expression in the developing gut is comparable to that in control embryo. (C) An example of embryo injected with exdpf morpholino (2ng of MO1exdpf). Note no strong GFP expression in the presumed pancreatic area at 24 hpf (arrow). A weak GFP positive pancreatic bud can be observed at 2 dpf (arrowhead). GFP expression in the gut is still comparable to that in the control and exdpf RNA injected embryos. (D–G) In situ hybridization using a ceruloplasmin probe with 3 dpf embryos. (D) A control embryo injected with standard morpholino control. (E–G) Examples of embryos injected with exdpf morpholino. Note comparable expression of cerulaplasmin in all embryos. (6.2 MB TIF) [file pbio.0060293.sg005.tif]

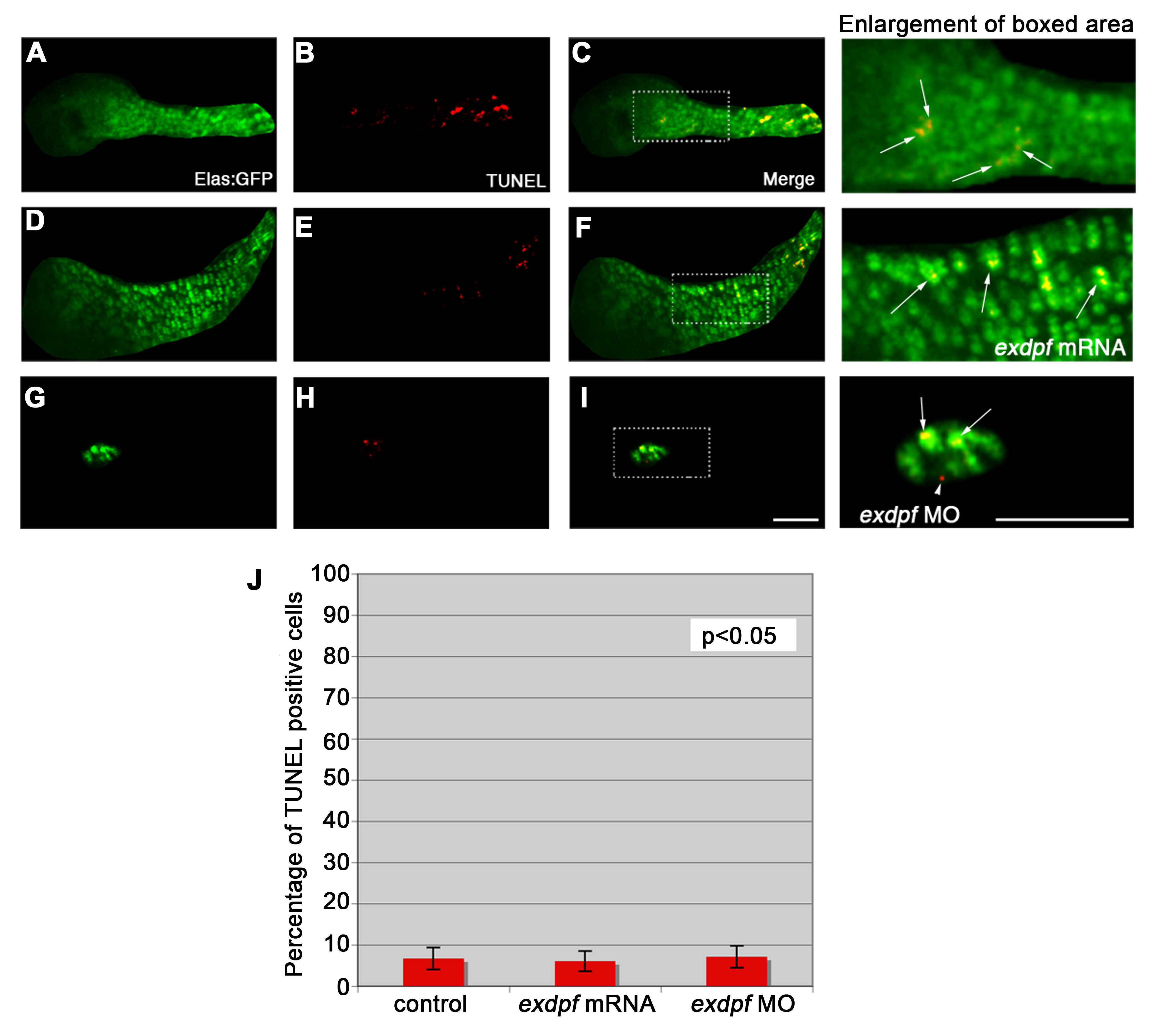

Supplement: Figure S6 — (TUNEL assay result in 5 dpf embryos. (A–C) A control embryo injected with standard morpholino control. Enlargement: of boxed area in (C). Note a few cell debris representing cells undergoing apoptosis (arrows in C enlargement). (D–F) An embryo injected with 100 pg of exdpf mRNA. Enlargement: of boxed area in (F). Note comparable number of cells undergoing apoptosis (arrows). (G–I) An embryo injected with exdpf morpholino. Enlargement: of boxed area in (I). Note both exocrine cells (arrows) and non-exocrine cells (arrowhead) undergoing apoptosis. Lateral view, anterior to the left. Scale bar: 50 μm. (J) A quantitative graph of TUNEL assay. (2 MB TIF) [file pbio.0060293.sg006.tif]

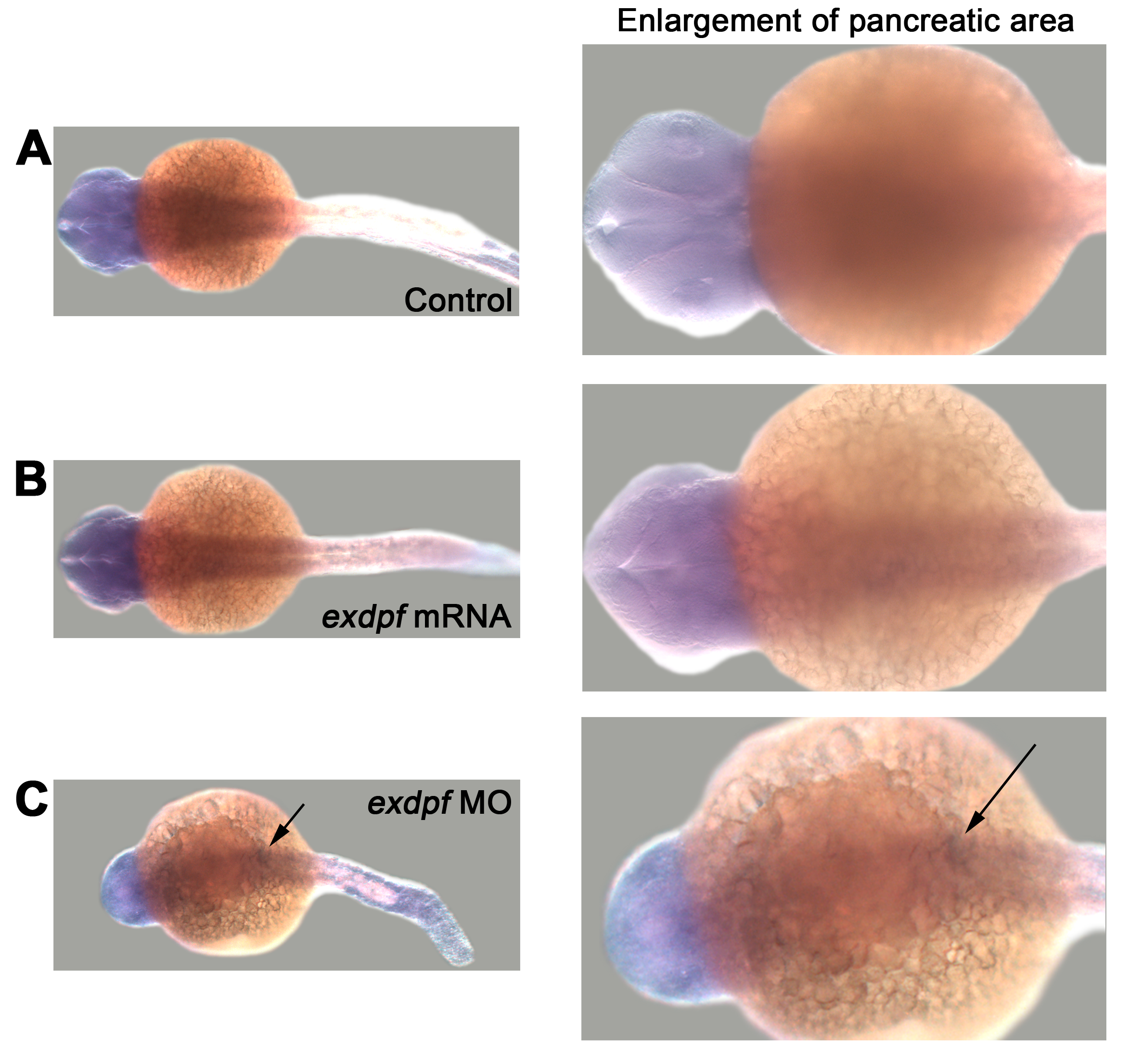

Supplement: Figure S7 — In situ hybridization using a p21 probe. (A) A control embryo injected with standard morpholino control. Note p21 expression in the head area. No p21 expression is detected in the pancreatic area. (B) An embryo injected with exdpf mRNA (100 pg). Note p21 expression in the head area. No p21 expression is detected in the pancreatic area. (C) An embryo injected with exdpf morpholino. Note p21 expression in the pancreatic area (arrow). (3.5 MB TIF) [file pbio.0060293.sg007.tif]

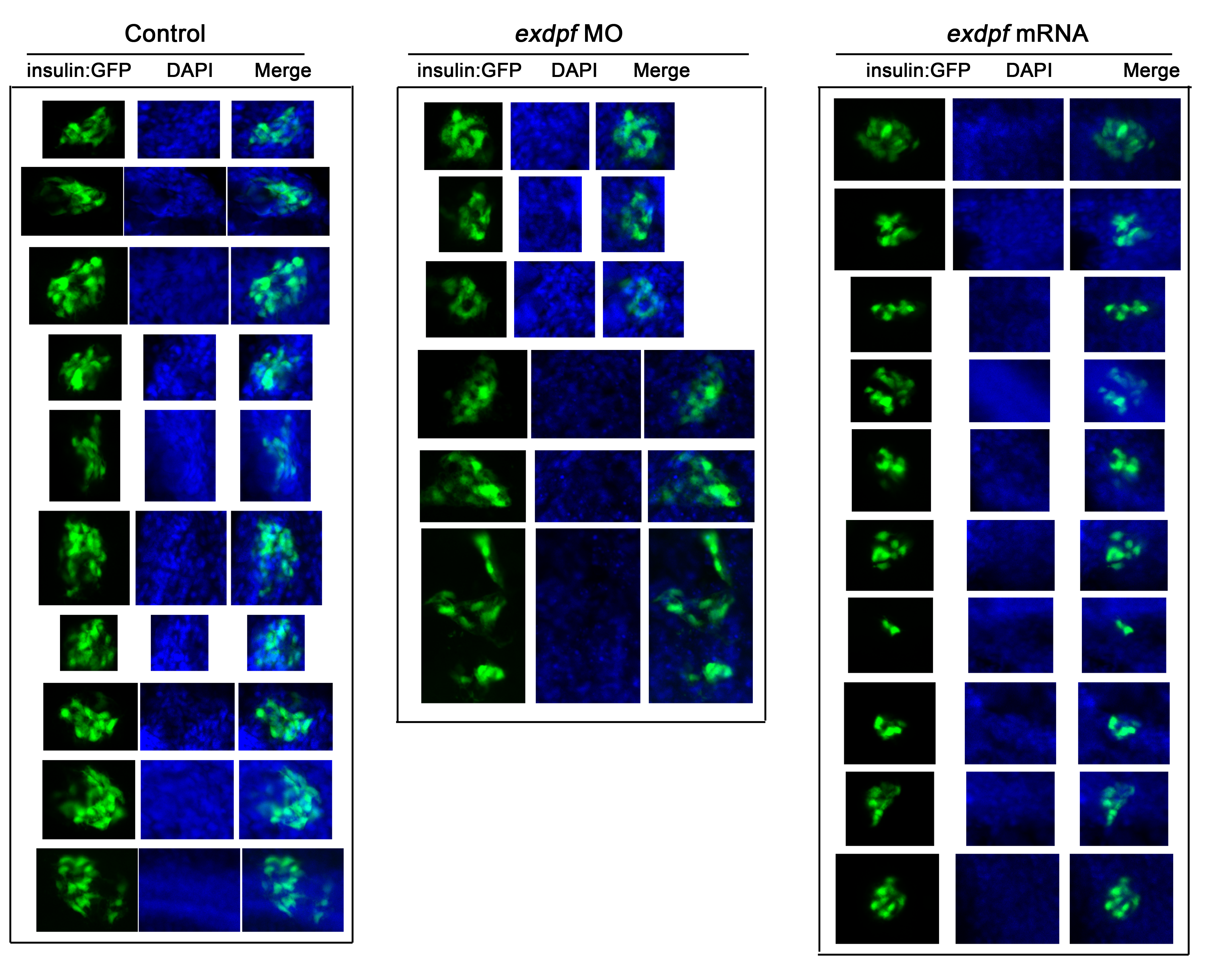

Supplement: Figure S8 — Green: preproinsulin:GFP. Blue: DAPI staining. All embryos are at 24 hpf, de-yolked and flat mounted. (6 MB TIF) [file pbio.0060293.sg008.tif]

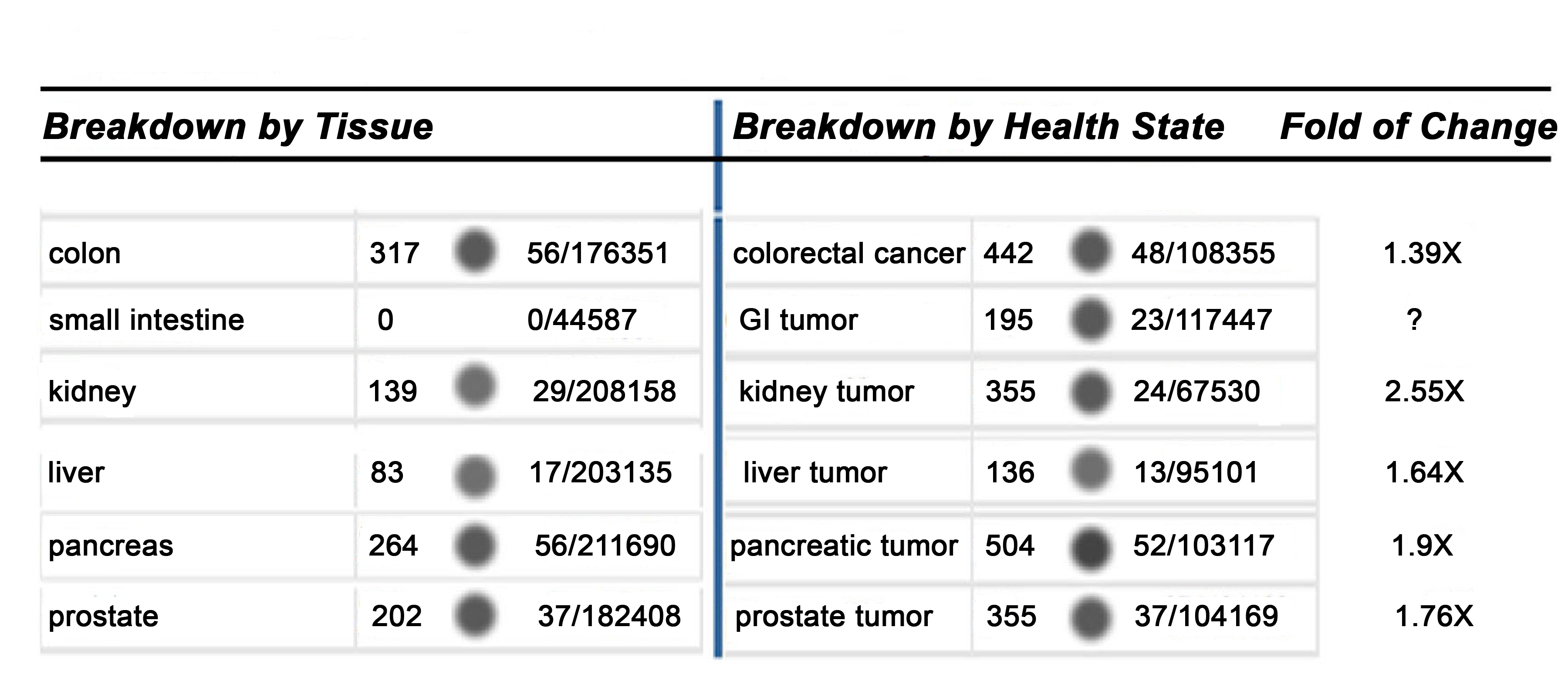

Supplement: Figure S9 — Human exdpf ortholog is expressed in different organs including colon, kidney, liver, and pancreas. Relatively higher level of exdpf has been detected in several tumors including colorectal caner, kidney tumor, liver tumor, and pancreatic tumor. (741 KB TIF) [file pbio.0060293.sg009.tif]
