# Supplementary material for: Exdpf Is a Key Regulator of Exocrine Pancreas Development Controlled by Retinoic Acid and ptf1a in Zebrafish
Source: PLoS Biol. 2008 Nov 25;6(11):e293. doi: 10.1371/journal.pbio.0060293 (PMC2586380; doi:10.1371/journal.pbio.0060293)
Supplement: Table S1 — The sequences of forward and reverse primers and product length for each gene tested are included. (27 KB DOC) [file pbio.0060293.st001.doc]

**Table S1. Primer pairs for RT-PCR.**

| **Gene** | **Forward** | **Reverese** | **Size** |
| --- | --- | --- | --- |
| P21 | GAAGCGCAAACAGACCAACAT | GCAGCTCAATTACGATAAAGA | 544 |
| P27 | CTGAAGCCTGGAACTTCGAC | TTATGTGGGTGTCGGACTCA | 372 |
| Cyclin G1 | GCCCTTTACAGTCCAGCCCAAATC | CTGTGCCTCAAGCCTCTCGATGTA | 500 |
| Cyclin D1 | GCTCGAGGTCTGTGAAGAGC | CTGACACGATCGCAGACAGT | 553 |
| EF1α | CTTCTCAGGCTGACTGTGC | CCGCTAGCATTACCCTCC | 358 |
